# Evaluating Double-Duty Actions in Rwanda’s Secondary Cities

**DOI:** 10.3390/nu16131998

**Published:** 2024-06-23

**Authors:** Sophia Demekas, Helen Prytherch, Francine Bayisenge, Straton Habumugisha, Klaus Kraemer, Jimena Monroy-Gomez, Immaculée Nabacu, Cornelia Speich, Innocente Turinimigisha, Dominique Barjolle

**Affiliations:** 1 Department of Environmental Systems Science, ETH Zürich, 8092 Zürich, Switzerland; sophwildem@gmail.com; 2 Swiss Tropical and Public Health Institute, 4123 Allschwil, Switzerland; helen.prytherch@swisstph.ch (H.P.); cornelia.speich@swisstph.ch (C.S.); 3 University of Basel, 4001 Basel, Switzerland; 4 Swiss TPH, Kigali Office, KG 501 St 9, Kigali 23WV+XP, Rwanda; 5 Sight and Life, Kigali Office, P.O. Box 325, Kigali 23WV+V3, Rwanda; straton.habumugisha@sightandlife.org (S.H.); nabacu.immaculee@sightandlife.org (I.N.); innocente.turinimigisha@sightandlife.org (I.T.); 6 Sight and Life, 4303 Kaiseraugst, Switzerland; klaus.kraemer@sightandlife.org; 7 Independent Researcher, 1700 Fribourg, Switzerland; lnjimenamonroy@gmail.com

**Keywords:** double burden of malnutrition, food system transition, nutrition policy, nutrition interventions

## Abstract

The double burden of malnutrition (DBM) is escalating in low- and middle-income countries (LMICs), including in Rwanda, most notably in urbanizing areas. The 2019–2020 Rwanda Demographic Health Survey (DHS) revealed that 33% of children under 5 years old are stunted while 42% of women in urban areas are overweight or obese. This coexistence has contributed to a surge in non-communicable diseases (NCDs), particularly in secondary cities. Using the World Health Organization’s (WHOs) “double-duty action” (DDA) concept, this study aims to identify and evaluate interventions with double-duty potential in Rwanda’s Rusizi and Rubavu districts and generate key recommendations for their improvement. A desk review of national policies pinpointed four programs with the greatest DDA potential: early childhood development (ECD) centers, the school feeding program, farmer field schools (FFS), and the provision of nutrition-sensitive direct support. In-person interviews with key stakeholders assessed the implementation of each program and a Strengths, Weaknesses, Opportunities, and Threats (SWOT) analysis was used to generate context-specific recommendations for their improvement. The main finding of this research is that Rwanda’s potential to address the DBM can be improved across multiple sectors by implementing a few key changes: targeting beliefs surrounding nutrition, improving trainings for community educators, enhancing parent–particularly father–involvement, and engaging in close monitoring and follow-up. These findings offer actionable streps that governments and nutrition stakeholders can take to improve similar interventions in other rapidly urbanizing LMICs.

## 1. Introduction

Known as the double burden of malnutrition (DBM), the co-existence of undernutrition (stunting, wasting, and micronutrient deficiencies) with overweight, obesity, and diet-related non-communicable diseases (NCDs) is a rising global threat, particularly in low- and middle-income countries (LMICs) [1]. Over the last 30 years, obesity rates have doubled globally and more than tripled in LMICs [2]. Today, LMICs attribute for over 85% of deaths from diet-related NCDs [3] and have a higher mortality from cardiovascular diseases (CVDs), diabetes, and chronic respiratory diseases than high-income countries [4]. One possible contributor to this is that nutrition policies in LMICs tend to be founded on “calorie fundamentalism”, meaning they prioritize increasing access to calories to reduce undernutrition [5]. Nutrition-targeted policies and programs are also often siloed and implemented by different governance and funding structures [6,7,8].

In an effort to remediate this, the World Health Organization (WHO) coined the concept of “double-duty actions” (DDAs) as cross-sector interventions, programs, or policies that target all forms of malnutrition simultaneously [9]. Health experts have since stressed the urgent need to prioritize research into DDAs moving forward given the current gap in research on their effects and areas for improvement [7,10].

### 1.1. DDA Conceptual Framework

The concept of DDAs is grounded in the notion that issues of under- and overnutrition share common drivers [6]:

1. Early life nutrition: Nutrition for pregnant and lactating mothers and children under 2 years has a profound influence on physical development for both mother and child and food-related behavior later in life;
2. Diet quality: Poor diet quality can lead to micronutrient deficiencies and/or excess consumption of nutrients-to-limit (e.g., salt, sugar, fat), both of which are associated with a higher risk of non-communicable diseases (NCDs);
3. Food environments: The way food is priced, marketed, and made available to a population all shape the food environment and influence food choices in a positive or negative way;
4. Socio-economic factors: Increased income is associated with lower rates of stunting and anemia but is simultaneously associated with a higher risk of overweight and obesity. Higher education is associated with lower rates of both.

**Figure 1:**
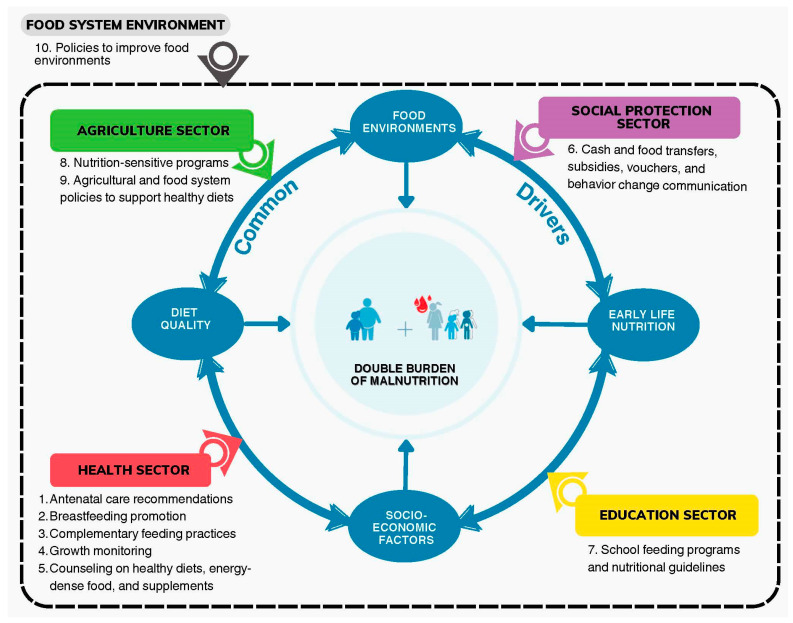
Ten priority candidates with double-duty action potential (numbered points) separated by sector (colored rectangles) targeted at the four common drivers (ovals) of the double burden of malnutrition (Source: Own elaboration).

In 2020, Hawkes et al. [6] introduced a framework to guide the design of DDAs that identifies ten priority entry points to target these shared drivers. Figure 1 lays out a new elaboration inspired by this initial framework, illustrating the ten priority entry points for DDAs across the sectors of health, social protection, education, agriculture, and the food system policy environment, which was adopted to guide the methodology of this paper (See Section 2).

### 1.2. Case Study: DBM and DDAs in Rwanda

Located in central sub-Saharan Africa, Rwanda is a small land-locked country known as the “land of 1000 hills”. While 74.5% of its land is used for agriculture, most of it is on hillsides with limited terracing and low irrigation [11]. Despite its small size, Rwanda is one of the most densely populated countries in Africa, with a population of 14.4 million, mostly concentrated in its central regions and its rapidly urbanizing Western province, where Rubavu and Rusizi are located [11,12]. Rwanda’s high population growth, limited land for agriculture, and vulnerability to an increasingly variable climate put its food system at significant risk and may lead to large sections of the country suffering from food insecurity and malnutrition [13].

While Rwanda has seen a considerable reduction in stunting and wasting over the past 20 years thanks to targeted nutrition policies [14], it has simultaneously experienced a steep rise in overweight and obesity [13]. Diet-related NCDs in Rwanda, such as diabetes, cancers, and CVDs, account for 44% of the country’s annual mortality and continue to rise at a concerning rate [3]. These trends are most severe in the Western province where food insecurity and malnutrition are the highest, particularly in secondary cities [13].

An upcoming report by the High Level Panel of Experts on Food Security and Nutrition (HLPE-FSN) on strengthening urban and peri-urban food systems has stressed the fragility of secondary cities’ food systems and emphasized the importance of strategic investments in food security and nutrition in these areas [15]. Rwanda’s Vision 2050, a development plan launched to spur economic growth, selected six secondary cities to invest in to ease the pressure of urbanization in Rwanda’s capital city, Kigali, and to distribute economic growth across the country [16]. Rubavu and Rusizi, two of the six secondary cities selected, are clear examples of urbanization’s exacerbating effects on the growing DBM.

In August 2021, the Nutrition in City Ecosystems (NICE) project, a multi-country and multi-stakeholder project aiming to improve healthy nutrition through sustainable, local food production in urban food systems [17], launched a baseline nutrition study in 150 urban households in Rubavu and Rusizi districts. The study confirmed that multiple forms and indicators of malnutrition were largely more severe in these urbanizing areas compared to the national averages [18]. For instance, compared to a national average of 33% stunting in children under 5, 28% of children under 5 surveyed in Rusizi were stunted as well as an alarming 47% in Rubavu [18,19]. The survey also found that overweight and obesity among women of reproductive age was higher in both Rubavu and Rusizi than at the national scale. Compared to 26% of women between 15 and 49 years in Rwanda, an alarming 42% in Rusizi and 45% in Rubavu reported being overweight and obese [18,20].

### 1.3. Objectives of the Paper

Given these results, this paper aims to assess Rwanda’s potential to combat the DBM by applying a framework based on Hawkes et al.’s [6] previous work to identify existing DDA programs active in Rwanda’s national policies and to evaluate how these are implemented at district level using Rubavu and Rusizi as case studies.

## 2. Methods

In a first step to identify the existing DDA entry points in Rwanda, a desk review of all nutrition-related policies and programs identified 12 ongoing interventions in Rubavu and Rusizi that qualify as DDAs. This was followed by stakeholder interviews to evaluate their implementation and to identify context-specific barriers to success and opportunities for improvement. The results of this analysis were analyzed and transformed into discrete recommendations for improvement for each program.

### 2.1. Data Collection

A desk review was conducted by searching the Global Database on the Implementation of Nutrition Action (GINA) and the Food, Agriculture, and Renewable Natural Resources Legislation Database (FAOLEX) for all nutrition-related policies and programs in Rwanda. Documents were considered nutrition-related if they included interventions targeting WHO’s 2025 Global Nutrition Targets: stunting, anemia, low birth weight, wasting, overweight, and breastfeeding practices [21]. If a relevant document was not up to date on either database, it was cross-checked on government ministry websites or obtained directly from government representatives via email request. If both the policy and strategic plan of the same document were available, the strategic plan was selected given its higher level of detail. National- and district-level plans, policies, and strategic plans written in English in their most recent published version were included while operational guidelines, regulations, standards, and legislative documents were excluded.

Twenty-four documents were considered nutrition-relevant and were read in their entirety. The objectives, outcomes, and activities in each were reviewed to identify ongoing projects that corresponded to the ten DDA entry points defined in Figure 1. Nine documents were excluded because they did not include DDAs, they had been replaced by an updated version, or because a Strategic Plan of the same policy was available. This left 15 documents and a total of 197 actions that qualified as DDA entry points (Supplementary File S1).

The 197 DDA entry points identified all corresponded to an active government or development partner-supported program (e.g., the national fruit tree program). Twelve of these programs were selected for further evaluation based on the following criteria: they were active in both Rubavu and Rusizi and the interviewees selected had direct experience in their implementation.

Key stakeholders were selected for in-person interviews using purposive sampling [22]. Participants were selected if they were professionals working in health, education, social services, or agriculture in Rubavu and Rusizi and were local collaborators of NICE. In total, 37 participants agreed to be interviewed, 17 in Rusizi and 20 in Rubavu, including district government officials, community health workers, schoolteachers, and members of non-governmental organizations (NGOs).

Table 1 lists the number and professions of interviewees selected from each district. Interviews were held between 27 June and 14 July 2023 and ranged from 19 min to 60 min. Interviews were conducted in English, French, or Kinyarwanda, depending on the preference of the participant, with the support of an interpreter familiar with the local context and culture. All interviews were recorded using a TASCAM Dr-05X (TASCAM, Zürich, Switzerland) audio recorder with written permission from participants and were manually transcribed afterwards in English. Each participant was provided with an information and consent form and a checklist of all DDAs of interest in both English and Kinyarwanda, after which they were asked to discuss the strengths and weaknesses of the ones they had experience in. Interviews followed a semi-structured open-ended question guide (Supplementary File S2) to allow participants the freedom to express their thoughts and allow certain responses to be questioned more in depth [23].

### 2.2. Data Analysis

QSR NVIVO was used to analyze transcripts via thematic coding [24] to extract common themes that fit within the four categories of SWOT analysis: strengths, weaknesses, opportunities, and threats. This analysis was selected given its recent recognition as a tool for incorporating community input into the design of health promotion strategies [25] and was adapted to this study to assess the DDA-relevant programs and develop actionable strategies for their improvement. Specific recommendations for improving each program were generated by fitting together themes across the four categories (S, W, O, and T) in an effort to address more than one at once [25].

### 2.3. Ethical Approvals and Considerations

This research project was approved by the Rwanda National Ethics Committee (FWA Assurance No. 00001973) on 16 June 2023 and the ETH Ethics Commission on 28 June 2023 as proposal EK 2023-N-128. All interviewees were informed that participation was voluntary and that they could choose to withdraw at any time or decline to respond to any question. All identifying information of participants was encrypted and safely stored on a password-protected computer. Results presented to focus groups and later published were presented in an anonymized form. There was no compensation provided for participation in this study.

### 2.4. Focus Group Discussion and Validation

Initial findings were discussed in focus groups with NICE project members and local program implementers for validation. Through follow-up discussions held over Zoom, the SWOT results for each intervention and resulting strategies were evaluated for technical and cultural validity. Following several rounds of feedback and discussion, final strategy recommendations were developed through consensus.

## 3. Results

### 3.1. Final DDA Interventions

Table 2 presents the twelve programs identified in Rwanda’s policies and strategic plans that show the greatest potential for a double-duty effect based on Hawkes et al.’s [6] conceptual framework and the selection criteria outlined above. Detailed descriptions of these programs are provided in Supplementary File S3.

### 3.2. SWOT Analysis Results

When asked to evaluate the successes and failures of the twelve programs in Table 2, participants provided their insights based on their lived experiences. These responses were combined and categorized into common strengths, weaknesses, opportunities, and threats in the form of one SWOT table for each program. All twelve SWOT tables are found in Supplementary File S4. For the purposes of this paper, we selected one program from each sector (health, education, social protection, and education), emphasized in bold in Table 2, for further analysis. The following four sections present the results of this analysis with a SWOT table for each program (P) and three recommendations for improvement for each. Recommendations for improvement for all twelve programs are found in Supplementary File S5.

- Program 1 (P1). Early Childhood Development (ECD) centers (Health Sector)

ECD centers provide services for children aged 3 to 6 years with goals to prevent delay in brain stimulation and ensure healthy growth in the period before they can attend primary school. Services include education, nutrition, protection, and sanitation and some centers also support pregnant and breastfeeding women through education sessions on proper childcare. ECD centers are jointly funded by the government, development partners and NGOs, and through parent contributions. As a result, centers vary significantly in the resources they are able to provide and in their ability to compensate caregivers, ranging from lowest capacity at home-based ECD centers to highest capacity at model ECD centers. Table 3 provides a SWOT matrix summarizing participant responses when they were asked to evaluate the benefits and challenges they faced with the ECD program in their district.

Interviewees unanimously spoke very highly of the impact ECD centers had in their community, both in improving child nutrition and in encouraging parents to adopt healthy practices in their homes. ECD caregivers expressed enthusiasm about the trainings they received, including how to prepare a balanced diet and how to start a vegetable garden. Common frustrations among participants were recurring food and material stock-outs, a lack of income for caregivers, and low parent contributions and involvement in the program. When asked how the program could be improved, interviewees suggested closer follow-up with parents to incentivize contributions and allowing the option to those who could not afford monetary contributions to contribute in other ways (e.g., by donating food, firewood, manure, or services). Others also brought up the idea of providing caregivers with incentives to attend trainings since, in most cases, income was infeasible.

Based on these responses, the SWOT matrix framework was used to generate specific recommendations for improving the program by fitting together themes across the four categories (S, W, O, and T) in an effort to address more than one at once [25]. Following, we present three recommendations generated for ECD centers by integrating relevant SWOT themes.

- P1-Recommendation 1. We recommend consulting with caregivers on which skills or topics they want training on and providing them with incentives to attend trainings, especially given that they are not compensated for their work at the ECD. Possible incentives could include transport allowances or free manuals and educational materials to take home.
- Relevant S, W, O, and Ts: S3; W2; O2, O4, O6, O7; T4
- P1-Recommendation 2. We recommend assigning specific responsibilities to parents, especially fathers, at ECDs to increase their involvement and thereby encourage them to adopt ECD services at home and motivate them to contribute. These responsibilities could include gardening, cooking, or cleaning tasks. We also recommend assisting parent groups to set up parent-led SILCs (Saving and Internal Lending Communities) through which they can collectively pool money to purchase materials for ECDs during stock-outs (e.g., porridge and milk).
- Relevant S, W, O, and Ts: S1, S2; W1, W2, W3, W4; O1, O3; T1, T2, T3, T5, T6, T7, T8
- P1-Recommendation 3. We recommend ensuring that all centers engage in at-home follow-up with households to ensure that parents are adopting ECD lessons on healthy diets and lifestyle practices at home.
- Relevant S, W, O, and Ts: S2; O5; T8, T9
- Program 2 (P2). Farmer Field Schools (Agriculture Sector)

The farmer field school (FFS) program recruits and trains farmer facilitators on “best agricultural practices” using local crops. Upon completion of the training, each FFS facilitator is assigned 25–30 farmers to educate in these practices and follow up with as well as a small plot of land in their community (demo plot) for demonstrations. Farmers are taught how to select breeds and healthy feed for livestock, maintain soil health, improve the quality of manure and organic fertilizer, and prioritize the planting of nutritious crops for at-home consumption, among other skills. Each step of the preparation, planting, maintenance, and harvesting process is demonstrated for the attending farmers. Table 4 summarizes participant responses on the benefits and challenges of the FFS program in the form of a SWOT matrix.

The FFS facilitators interviewed agreed that hands-on model plot demonstrations and close follow-up with farmers, either in-person or through regular SMS reminders, were effective at motivating farmers to adopt and follow new practices. A challenge that facilitators repeatedly faced was the high cost of inputs and poor quality of feed on the market, which discouraged farmers from adopting and following new practices. Another challenge was the common practice of selling production, particularly livestock or animal-based products, to buy more land or expensive inputs. These challenges were exacerbated in cases when facilitators were unable to follow up with farmers over long periods of time. When asked how the program could be improved, facilitators requested more frequent trainings and recruitment of facilitators.

- P2-Recommendation 1. We recommend investing in establishing a local feed supplier and distributor in or accessible to Rubavu and Rusizi districts with the capacity to supply high-quality feed in sufficient quantities to surrounding farming households. We also recommend incentivizing or supporting research and education on sustainable feed alternatives as some FFS facilitators have already accomplished with black soldier flies.
- Relevant S, W, O, and Ts: W2; O1; T3, T4, T6
- P2-Recommendation 2. We recommend increasing the number and frequency of trainings and providing regular refresher sessions for FFS facilitators to sustain their motivation and encourage continuous learning.
- Relevant S, W, O, and Ts: S2, S3; W3; O3
- P2-Recommendation 3. Given that in-person follow-up to all farmers by facilitators is often infeasible, especially in rural areas, we recommend scaling up SMS reminders for farmers to ensure they are implementing the lessons they learned at appropriate times (e.g., weeding, harvesting, and cover-cropping) and encourage them to not sell their production.
- Relevant S, W, O, and Ts: S1; W1, W3; O2; T1, T2, T5
- Program 3 (P3). School feeding program (Education Sector)

The aim of the school feeding program is to ensure that every child in Rwanda receives at least one healthy meal per day if they attend school. The average cost of a base meal for one child is 150 RWF and, while the government subsidizes some of this cost (56 RWF), the child’s parents must cover the rest. Parents who are unable to contribute money can contribute food, materials, or services instead. Some schools have gardens run by teacher-led nutrition clubs that are used to grow vegetables intended to supplement school meals. Table 5 provides a SWOT matrix summarizing participant responses when they were asked to evaluate the benefits and challenges that they faced with the school feeding program.

The teachers and school administrators we interviewed emphasized the significant benefits the school feeding program has had at improving school attendance and focus in class since its inception. Common challenges they faced were the lack of animal products, fruits, and diverse vegetables in school meals due to low parent contributions. They explained that some parents regard the school feeding program as the responsibility of the government and are discouraged from contributing because they do not trust the motives of the teachers and the school system. Other shared frustrations were insufficient cooking materials to meet demand and a lack of designated eating spaces for children, leading to unhygienic classrooms and disruption during class time. When asked how the program could be improved, interviewees suggested scaling up the school garden program and building trust among parents to incentivize higher contributions.

- P3-Recommendation 1. We recommend investing in building or finding designated eating spaces at schools and in acquiring sufficient kitchen and cooking materials to meet demand.
- Relevant S, W, O, and Ts: O4; T3, T5
- P3-Recommendation 2. We recommend increasing collaboration between parents, local leaders, and schoolteachers to help cultivate trust and, as with the ECD program (see program 1), establishing SILCs to help parents purchase fruits, vegetables, and animal products for school meals and allow them to gain transparency on how and where their contribution is spent.
- Relevant S, W, O, and Ts: S1, S2; W1, W2; O1, O2; T1, T2, T4
- P3-Recommendation 3. We recommend establishing links between schools and FFSs for local procurement and as a resource for agricultural education sessions. By linking the two programs, FFS facilitators could use school gardens as “model plots” for their farmer trainings and the resulting production could be used to improve the quality and diversity of school meals. We also recommend extending the option for parents and teachers to attend some of the trainings when possible to encourage their involvement at schools.
- Relevant S, W, O, and Ts: W1, W2; O3; T1, T4
- Program 4 (P4). Nutrition-Sensitive Direct Support and Shisha Kibondo (Social Protection Sector)

Nutrition-Sensitive Direct Support (NSDS) is a cash transfer scheme targeting poor households with pregnant women and/or children under 2 years to incentivize them to access essential health and nutrition services. Shisha Kibondo, a fortified blended foods (FBF) that is produced locally, is a maize-corn blend with vitamin/mineral premix provided to pregnant women from vulnerable households or mothers who have a child identified as malnourished for at-home preparation [26]. Table 6 summarizes participant responses on the benefits and challenges of these social protection programs in the format of a SWOT matrix.

A major concern that interviewees raised about NSDS is that, given that mothers are only eligible for the cash transfer during pregnancy and up until their infant reaches 2 years of age, they may be incentivized to become pregnant again to avoid losing the nutritional support. A common criticism among interviewees about the Shisha Kibondo program was the inability to ensure that, after distribution, the product is being used properly. Many beneficiaries choose to sell the product instead of feeding it to their malnourished child or dilute it to share among all the children at home. As a result, malnourished children in families with many siblings rarely see the intended effects of Shisha Kibondo. When asked how the program could be improved, interviewees suggested conducting close follow-up to ensure Shisha Kibondo was being administered properly.

- P4-Recommendation 1. To address the concerns surrounding NSDS about incentivizing women to become pregnant, we recommend complementing any monetary support to beneficiaries with targeted education about family planning. We also recommend extending the support until the child reaches three years of age instead of two, at which point children are generally eligible to attend ECDs.
- Relevant S, W, O, and Ts: S1; W1; O2; T1, T4
- P4-Recommendation 2. Currently, Shisha Kibondo is distributed at community health centers at regular growth monitoring sessions for women to take home and administer themselves. We recommend linking the Shisha Kibondo program to ECD centers by training caregivers to administer Shisha Kibondo at ECDs. This could both ensure that mothers and children receive adequate quantities of the product and additionally incentivize pregnant and lactating women to attend ECDs where they could receive other antenatal and postnatal services and counseling. After the first initiation at ECDs, beneficiaries could begin to receive it again for at-home administration but with close and regular follow-up.
- Relevant S, W, O, and Ts: S1; W1, O1, O2, O3; T2, T3
- P4-Recommendation 3. Currently, the quantity of Shisha Kibondo provided to beneficiary households is based on the estimated quantity needed for only one child. We recommend calculating the quantity of Shisha Kibondo provided based on household size (i.e., number of children or household members) instead. This could reduce the likelihood of beneficiaries diluting the product to meet the needs of all family members.
- Relevant S, W, O, and Ts: W1, O2, T3

## 4. Discussion

The four programs evaluated in this paper represent four entry points to tackle the double burden of malnutrition in Rwanda in four key sectors: health, agriculture, education, and social protection. Although our recommendations are specific to each program and are based on the specific experiences of the interviewees, some common themes are apparent across all evaluated DDA programs in Rubavu and Rusizi and prove consistent with other research.

First, interviewees often cited a lack of physical materials, space, or capacity as a barrier limiting an intervention’s success. Insufficient or poor-quality feed for FFS (P2) and insufficient kitchen capacity at school kitchens (P3), for instance, hindered the success of these programs and we recommend NICE and other development partners prioritize procurement of necessary materials as a first step towards improving their implementation. In a 2021 scoping review by Ezezika et al. exploring the barriers and facilitators to the implementation of large-scale nutrition interventions in Africa, ineffective resource mobilization was identified as one of the major barriers to scaling up nutrition initiatives, with material stock-outs as a driving factor [27].

Second, a commonly cited barrier among participants was that of shared beliefs concerning nutrition and food, including that nutrition programs should be the responsibility of the government (P3), that healthy food is only for the wealthy (P2), and that quantity of nutrition outweighs quality (P4). Previous household assessments conducted in Rwanda in 2019 have confirmed this in their finding that optimal feeding and physical activity practices among children were limited because of cultural beliefs that overweight in children is a sign of good health and that fruits are only meant for wealthy families [28]. Another theme identified by Ezezika et al.’s scoping review as a barrier to program implementation was a lack of cultural understanding and adaptability from beneficiaries [27]. In order to build trust in the community, we recommend targeting social behavior change campaigns (SBCCs) to address specific beliefs surrounding nutrition. SBCCs have proven to be effective in the past at improving dietary practices when combined with nutrition interventions in Rwanda [29] as well as in nutrition interventions targeting malnutrition, micronutrient supplementation, and infant and young child feeding practices (IYCF) in multiple other countries [30].

Third, community educators including teachers, caregivers, FFS facilitators, and community health workers (CHWs) frequently expressed a desire for more trainings to further their education. This was consistent with the findings of another qualitative assessment conducted in 2014 in Rwanda which found that most CHWs found trainings important and helpful but felt that they were too inconsistent and insufficient [31]. Ezezika et al.’s scoping review highlighted training and support for facilitators, such as refresher courses and targeted mentoring, as important enablers for implementation [27]. We recommend increasing the frequency of trainings for caregivers and CHWs on topics of their choice (P1), providing regular refresher trainings for FFS facilitators (P2), and training ECD caregivers to administer Shisha Kibondo (P4). As crucial players in educating the population about nutrition and health [32], investment in enhancing the knowledge, motivation, and even empowerment of these actors is a critical step in overall program improvement.

Fourth, interviewees often explained that nutrition programs were most effective when community members were directly involved and felt a sense of ownership and autonomy in the program. Previous evaluations of agricultural interventions in Rwanda have shown that they significantly benefited from the formation of agricultural cooperatives because they cultivated a sense of ownership and responsibility within the targeted community [33]. SILCs and similar mechanisms of microfinance, such as village savings and lending associations (VSLAs) have also been researched in other contexts and been shown to increase adherence to maternal health interventions and contribute to improved child nutrition and health, particularly when women were the participants [34,35]. We recommend assigning parents roles and responsibilities at ECD centers (P1) and schools (P3) and assisting them in forming their own SILCs as ways to engage program beneficiaries and to increase the program’s chance of becoming sustainable without government or donor support.

Fifth, interviewees across all sectors highlighted the importance of close follow-up with program beneficiaries to ensure lessons were being followed and best practices implemented at home. A cluster-randomized trial conducted in Rwanda in 2021 found that Sugira Muryango, a home-visiting intervention involving regular follow-up sessions with beneficiaries, significantly increased adoption of ECD practices at home [36]. Similarly, Ezezika et al.’s scoping review found that routine monitoring and evaluation were crucial to the implementation of nutrition programs [27]. We recommend scaling up at-home follow-up sessions by ECD caregivers (P1) and regular SMS reminders by FFS facilitators (P2), for instance, as concrete steps to incorporate monitoring and follow-up into these respective programs. Importantly, these steps would have to be taken with a specific plan in mind with whom the results of this monitoring would be shared as well as how they would be used for future program improvement.

As illustrated in Figure 1, DDA interventions targeting DBM’s shared drivers are still siloed by sector with different authorities responsible for their implementation [7]. Moving forward, it will be important to find synergies between DDAs across sectors to ensure they are not working in isolation or in conflict with one another. Linking schools and FFS for procurement and training purposes (P2 and P3) and training ECD caregivers how to administer Shisha Kibondo (P1 and P4) are examples of how programs can be linked to engage multi-stakeholder collaboration for mutual benefit.

Finally, it must be acknowledged that the DDA interventions in Rwanda are almost all designed to target women specifically. Shisha Kibondo, ECDs, and NSDS, for example, all directly target pregnant women or mothers. Even agricultural interventions and educational interventions, like the school feeding program, focus more on engaging women in the community than men. Formative consumer research in Rwanda has shown that the cultural norm considers all food-related activities as a woman’s duty [37]. Purchasing food, preparing meals, and feeding the family are generally roles assigned to women in the household. However, men in Rwanda are considered the heads of households and hold the final purchasing and decision-making power, thus having a large influence over nutrition and dietary practices at home. Importantly, qualitative assessments of gender roles in nutrition interventions have found that men in Rwanda are interested in learning about nutrition and in receiving training, particularly in male-only spaces [37]. This introduces an important question for further investigation: what can be done to increase the involvement of men in DDA nutrition interventions? While addressing this issue in depth exceeds the scope of this study, it is important to mention that all four programs discussed can be improved by targeting the involvement of fathers and men, particularly in educational and behavior change efforts.

### Strengths and Limitations

This study provides a rare glimpse into local perspectives and insights from an area where qualitative data coverage is generally limited and adds a valuable piece of research to the sparse literature on DDAs and their implementation [38]. Several key limitations must be acknowledged. First, the selection of programs for evaluation and the relative importance given to the strengths, weaknesses, opportunities, and threats documented were biased by the experiences of the participants interviewed. Second, purposive sampling to select participants is prone to bias as it depends on the researcher’s subjective judgment of which professions are most relevant to the study. A SWOT analysis similarly depends on the researcher’s judgment in categorizing which factors fall into the four SWOT categories. Another limitation is the possible loss of meaning or context during transcription given that answers were first orally translated from Kinyarwanda to English. Lastly, the focus group discussions were limited by time constraints and low availability of group members. Outside the scope of this study, more rounds of discussion and validation would be necessary to ensure the feasibility, cultural appropriateness, and relevance of the strategies proposed in this paper.

## 5. Conclusions

Rubavu and Rusizi, two secondary cities in Rwanda’s Western province, suffer from higher rates of stunting and overweight and lower dietary diversity than the national average [20]. Rapidly urbanizing areas in LMICs tend to be most at risk of DBM due to contradictory nutrition policies and siloed governance, compounded by a lack of proper infrastructure and physical capacity [7,39].

This study aimed to evaluate the potential for DDAs in Rwanda’s nutrition policies and programs to help tackle the growing DBM in Rubavu and Rusizi, two rapidly urbanizing areas compounded by multiple forms of malnutrition. Through a desk review and subsequent in-person interviews, we identified twelve programs in Rubavu and Rusizi–four of which were discussed in this paper–with DDA potential and evaluated their local implementation to identify areas for improvement. Findings were summarized into strategy recommendations which were thematically grouped across all interventions into seven strategic actions.

First, we recommend increasing access to the basic physical materials and building the capacity necessary for program implementation. Second, we suggest incorporating mobilization and education components into all interventions to target culturally shared beliefs related to nutrition. Third, we recommend improving the quality of interventions by ensuring community educators attend recurring and reliable trainings. Fourth, we recommend designing interventions to be self-sustaining by increasing community involvement to build a sense of ownership and responsibility. Fifth, we recommend all interventions have a plan for follow-up after program initiation and monitoring the impact after uptake. Sixth, we encourage merging different DDA interventions where there are synergies to avoid siloed governance. Lastly, we recommend taking measures across all interventions to ensure a more balanced involvement of men and women in matters of household and community nutrition.

Looking forward, we hope these findings spur future investigation, within the NICE project or externally, into how to design SBCCs and community mobilization strategies that target shared beliefs surrounding nutrition while remaining culturally sensitive and locally appropriate and, particularly, how to appeal to and encourage the involvement of men.

## Figures and Tables

**Table 1:** Number of interviewees by profession in Rusizi and Rubavu.

Sector	Position	Rusizi (17)	Rubavu (20)
Health	District Directors of Health	1	1
	Nutritionists in district hospitals	1	1
	Antenatal care (ANC) providers and nurses	1	1
	Community health workers (CHWs)	1	1
	Early Childhood Development (ECD) center workers: leaders, caregivers, and volunteers	2	3
Education	District Directors of Education	1	1
	Schoolteachers (Heads of Nutrition and Environment clubs)	3	2
Social Protection	ECD District Focal Points	1	1
	National Women’s Council representatives	1	1
	Members of non-governmental organizations (NGOs)	2	2
	Representatives of faith-based organizations (FBOs)	0	2
Agriculture	District Directors of Agriculture	1	0
	Farmer Field School (FFS) facilitators	1	2
	One Acre Fund officers	1	1
	Veterinarians	0	1

**Table 2:** Desk review results: 12 DDA programs by sector.

Sector	DDA Program
Health Sector	1. Antenatal care (ANC) visits
	**2. Early Childhood Development (ECD) centers**
	3. Exclusive breastfeeding promotion
Education	**4. School feeding program**
	5. Trainings (teachers, caregivers, and community health workers)
Social Protection	6. Social behavior change campaigns
	7. Non-communicable disease (NCD) prevention and physical activity promotion
	**8. Nutrition-Sensitive Direct Support (NSDS) and Shisha Kibondo**
Agriculture	9. Farmer Field Schools (FFS)
	**10. Small stock distribution**
	11. Fruit tree program
	12. Kitchen garden program

Note. **Bold** indicates the program was selected for further evaluation.

**Table 3:** SWOT: Early childhood development (ECD) centers.

**Strengths**	**Weaknesses**
S1. Parents see changes in children and are motivated to follow the ECD model at home S2. Home visits show that parents start implementing kitchen gardens after seeing them at ECDs S3. Training of ECD caregivers improves quality of services and trainings	W1. Low parent monetary contribution W2. Inconsistent and unequal support across ECDs (meals, materials, and caregiver support) W3. Not enough food at ECDs for all children W4. Lack of fruits and animal products in meals provided at ECDs
**Opportunities**	**Threats**
O1. Option for parents to make non-monetary contributions (food; firewood and charcoal; cooking; cleaning; and gardening services) O2. Increased trainings for caregivers (ensuring continuous education) O3. Increased parent involvement (e.g., parents form groups to raise money to improve ECDs) O4. Income or non-monetary compensation for caregivers (money is best incentive even if small amount) O5. Close follow-up (home visits to monitor changes or adoption of practices) O6. Incentives for caregivers to attend trainings O7. Introduce attractive new topics for trainings based on what caregivers want to learn	T1. Shared mindset that ECDs are a government program and responsibility T2. High cost of healthy food at market and high price volatility T3. Lack of sufficient funding T4. Caregivers not compensated or incentivized to attend trainings and many do not have the time T5. Stock-outs (porridge stock-outs lead to drop-outs or ECD closures) T6. Lack of long-term program sustainability without partner or government support T7. Inconsistent government budget (leads to stock-outs) T8. Lack of knowledge (parents do not know or value proper ECD services) T9. Parents do not have the time to attend ECD trainings or education sessions

**Table 4:** SWOT: Farmer Field Schools (FFS).

**Strengths**	**Weaknesses**
S1. SMS reminders for farmers (when to top dress, weed, and harvest) work well when in-person follow-up is impossible S2. Model plot hands-on demonstrations are effective at engaging farmers S3. Farmers reached through the program are motivated to learn new methods	W1. Animal products are the first thing farmers sell on the market (not kept for at-home consumption) W2. Lack of feed industry in many districts results in high costs and long wait times to acquire feed W3. Often there are not enough trained FFS facilitators to cover all farmers
**Opportunities**	**Threats**
O1. Improved quality of feed for small stock (alternative methods, e.g., black soldier flies) O2. Continuous education for farmers and follow-up from FFS facilitators O3. More frequent and recurring trainings for FFS facilitators on best practices and on facilitation	T1. Farmers prioritize selling production to buy land or other inputs rather than consuming it T2. Farmers who do not receive follow-up become disincentivized to continue implementing best practices T3. Inputs and feed are expensive on the market (organic fertilizers especially) T4. Shared mindset to value quantity of production over quality T5. Belief that nutritious food is for wealthy people T6. Small stock often fall ill (due to bad practices or poor quality of feed)

**Table 5:** SWOT: School feeding program.

**Strengths**	**Weaknesses**
S1. Non-monetary contributions by parents can help subsidize school meals (e.g., small fish, peanuts, and sweet potatoes) S2. Parent meetings and mobilization increase awareness, motivation, and contributions S3. Daily school meals have improved school attendance and focus in class	W1. Low parent contribution W2. Lack of animal products or vegetables or fruits in the menu (always maize, beans, rice, potatoes, and soya)
**Opportunities**	**Threats**
O1. Increase collaboration between parents and school to increase contribution and involvement O2. Increase collaboration with local government or leaders to encourage parents O3. Run trainings at schools for parents on starting and maintaining vegetable gardens O4. Increased kitchen and eating materials to meet demand (e.g., “muvero”, plates, utensils, and cooking needs)	T1. Parents, especially those with many children, are unwilling or cannot afford to contribute to the program (contribution is per child) T2. Mindset that school feeding is government’s responsibility T3. No specific room for eating (refectory) T4. Lack of trust or respect for teachers or school feeding committee T5. Lack of kitchen materials (makes food preparation time consuming)

**Table 6:** SWOT: Nutrition-Sensitive Direct Support (NSDS) and Shisha Kibondo.

**Strengths**	**Weaknesses**
S1. Stunting prevention measures taken before birth and continued into childhood	W1. Big families with many children rarely see effects of Shisha Kibondo as they are often distributing the Shisha Kibondo among all children in the household rather than only serving to the malnourished child
**Opportunities**	**Threats**
O1. Close monitoring through home visits to ensure Shisha Kibondo is properly prepared and consumed O2. More holistic support to vulnerable family instead of to just one child O3. Administer Shisha Kibondo outside the home, e.g., directly in health facilities or ECDs	T1. Provides contradictory messaging to family planning interventions T2. Beneficiaries sell Shisha Kibondo rather than consuming it T3. Beneficiaries dilute and share Shisha Kibondo among many children at home T4. Forgotten age group (support lasts only until 2 years)

## Data Availability

The original contributions presented in this study are included in this article/supplementary material, further inquiries can be directed to the corresponding author.

## Supplementary Materials

The following supporting information can be downloaded at: https://www.mdpi.com/article/10.3390/nu16131998/s1.
