# Supplementary material for: Evaluating Double-Duty Actions in Rwanda’s Secondary Cities
Source: Nutrients. 2024 Jun 23;16(13):1998. doi: 10.3390/nu16131998 (PMC11243673; doi:10.3390/nu16131998)
Supplement: Supplementary file 1 [file nutrients-16-01998-s001.zip › Supplementary File S1.pdf]

Appendix A. Desk Review Results: DDA-relevant actions

**Fourth Health Sector Strategic Plan 2018-2024**						
**Priorities by Services, Programs, and Health Systems**	Health Services	Social Safety Nets	Educational Setting	Agriculture	Food Environments	10 Priority Candidates
**Chapter 4.1 Health Services and Programs Across the Life Cycle**						
**4.1.1 MCCH (Pregnancy, Early Life, and Children)**						
Improve and sustain quality of MCH (maternal child health) services	X					1,3
Increase ANC and postnatal care uptake	X					1
**4.1.2 Nutrition**						
Community education and awareness on dietary and complementary feeding practices	X					3
Establishment and use of ECDs as entry point of provision health interventions (specifically nutrition)	X					1,3,4,5
Prevention and management of malnutrition (acute and chronic)	X					1,3,4
**3.4.1 Strategies for mainstreaming NCDs in various sectors**						
**a. Education Sector**						
Integrate NCD prevention and control in school curricula at various levels of education	X		X			5,7
**e. Agriculture Sector**						
Ensure production of healthy food crops (fruit, vegetables, cereals and other sources of healthy food)				X		8,9
**h. Trade and industry sector**						
Regulate the trade of processed food and beverages		X			X	6,10
**Maternal Newborn and Child Health Strategic Plan 2018-2024**						
**Key Priorities by Strategic Intervention**	Health Services	Social Safety Nets	Educational Setting	Agriculture	Food Environments	10 Priority Candidates
**Strategic Objective 1. Universal access to quality MNCH services for all citizens**						
**Key Implementation Priorities**						
Maternal and child nutrition: maternal pre- and during pregnancy and post-partum; child nutrition especially between 6-24 months	X					1,2,3,5
Stunting prevention: coordinate with the National Food and Nutrition Secretariat on implementation priorities (promotion and implementation of services, interventions and practices that prevent stunting during the 1st 1000 days of life; micronutrient supplementation; timely identification and treatment of undernutrition)	X					1,2,3,4
**Strategic Objective 3. Enhanced community health literacy, skills and practices through social, behavioral and community engagementn efforts to improve equitable MNCH**						
**Key Implementation Priorities**						
Complementary feeding among 6-24 months	X					3
**Strategic Objective 4. Available, accessible, acceptable and high quality MNCH workforce developed and enforced at all levels of service delivery**						
**Key Implementation Priorities**						
Maternal, newborn and child nutrition	X					1,3
Antenatal care (8 contacts) - including counseling and care in early pregnancy	X					1
**National Agriculture Policy 2018**						

Outcomes and Policy Actions by Pillar	Health Services	Social Safety Nets	Educational Setting	Agriculture	Food Environments	10 Priority Candidates
**Pillar 2: Technological Upgrading and Skills Development**						
**Outcome: Technological upgrading and capacitated farmers and rural value chain actors who make informed decisions and profitably engage in off-farm activities**						
2.3 Promote research on nutrient-rich crops through bio-fortificaiton, to position Rwanda as a trusted source of bio-fortified food for the region				X	X	9,10
2.19 Promote research on diversified animal products				X		8
2.29 Focus on domestic market research on identifying dietary gaps, mapping consumptIon patterns and the links with production, trade analysis, impact of regulations				X	X	8,10
2.44 Broaden the public extension services to include business orientation, nutrition, gender and savings, using weather and climate information, IMP and climate smart agriculture				X		8
2.52 Provide targeted support to women to access suitable financial products for income-generating on-farm and off-farm activities				X		8
2.53 Support access to start-up funds for women to start or grow their agribusiness enterprises				X		8
**Pillar 4. Inclusive Markets and Off-Farm Opportunities**						
**Outcome: Improved inclusiveness of agricultural market systems and increased value addition and competitiveness of diversified agriculture commodities for domestic, regional,**						
4.10 Develop household nutrition guidelines	X					5
4.11 Develop practical skills training from local Community Health staff, extensionists, and Farmer Field Schools	X			X		3,8
4.12 Expand and revise kitchen garden programs				X		8,9
4.13 Scale up the availability of bio-fortified foods					X	10
4.14 Coordinate with social protection programs to asset building mechanisms (distribute small livestock, access to inputs)		X		X		6,8
4.15 Promote local procurement of diversified nutritious foods for school meals			X			7
4.16 Mainstream and coordinate efforts to identify and address specific dietary gaps through direct investments in agriculture and rural development				X		8
4.20 Expand the body of publicly available data on prices and production and facilitate market linkages between smallholder farmers, cooperatives, traders, and the processing industries					X	10

National Comprehensive School Feeding Policy 2019

Recommended Policy Actions	Health Services	Social Safety Nets	Educational Setting	Agriculture	Food Environments	10 Priority Candidates
**5.1 Scale up the coverage of school feeding programs**						
Universal coverage of school feeding programs for pre-primary, primary, and secondary school children (including private schools, which prior was not the case past primary school) thorugh increased government subsidies and parents' contribution			X			7
Vulnerable children (e.g. orphans and OVC) and children from vulnerable households (Ubudehe 1 and 2) are exempt from parent requirement to contribute for feeding			X			7
**5.2 Ensure health and nutrition sensitive school feeding programming**						

MINEDUC will work with Nutrition and Food Technologist to develop a guide on a school menu that ensures nutritious meals with sufficient macro and micronutrients			X			7
Guidelines on how to develop appropriate food menu (include fresh food in food baskets) will be developed by MINEDUC			X			7
School feeding program accompanied by comprehensive package of complementary interventions: water, sanitation, hygiene facilities; health, nutrition, hygiene education; systematic deworming; micronutrient supplementation; psychosocial support; energy saving stoves and clean cooking			X			7
MINEDUC to develop guidelines for minimum requirements for school feeding requirements (infrastructure and equipment required)			X			7
**5.3 Promotion of school gardening and farming**						
School gardening and farming programs will act as educational forum for children and parents and increased production for school feeding			X	X		7,8
MINAGRI will define minimum package and provide support to school gardening and farming			X	X		7,8
Parents of targeted school children will be involved in the management of school gardens and farming			X	X		7,8
Food production from gardens/farms will be added to school feeding basket when available			X	X		7,8
**5.5 Create appropriate policies and frameworks linking market access to farm produce by local farmers to the school feeding programs using unconventional procurement**						
School feeding programs will be linked to locally produced food for long-term food and nutrition security and community development			X	X		7,9
MINEDUC will develop appropriate supply chain or procurement model with detailed guidelines to ensure transparent, efficient and accountable approach from local farmers using unconventional procurement method			X	X		7,9
**National Early Childhood Development Policy (NECDP) Strategic Plan 2018-2024**						
**Key Strategic Interventions by Priority Area and Pillar**	Health Services	Social Safety Nets	Educational Setting	Agriculture	Food Environments	10 Priority Candidates
**Strategic Direction 2. Improved and sustained quality health and nutrition status of infants and young children in first 1000 days of life**						
**Outcome 2. Increased, equitable access to high impact, evidence-based health, nutrition, family planning and reproductive health services at primary and community level to**						
1. Promote 4-8 antenatal care visits and integrate/strengthen surveillance and prevention of malnutrition as part of antenatal care	X					1,4
3. Support for early initiation and exclusive breastfeeding	X					2
4. Support continued breastfeeding up to 24 months and appropriate complementary feeding	X					2,3
6. Establish and strengthen mechanisms to address anaemia among adolescent girls	X					5
7. Strengthen growth monitoring and promotion at health facility and community levels, including Intervention and referral	X					4
8. Roll-out the child length mat to support visualization and early detection of stunting	X					4

9. Strengthen the quality and coverage of Maternal Infant and Young Child Nutrition counselling (individual and group counselling) through existing opportunities such as ANC (antenatal care), PNC (post-natal care), immunization sessions, and IMCI (Integrated Management of Childhood Illness), targeting all care providers (mothers, fathers and caretakers)	X					1,2
10. Build capacity of health care providers for identification, quality care/treatment, referral, and follow up after discharge of moderate and severe acute malnutrition	X					4
14. Improve nutrition practices and education in schools (primary & secondary) and ECD facilities			X			7
**Strategic Direction 4. Improved and sustained equitable food security for family health in vulnerable households with pregnant and/or lactating women**						
**Outcome 4. Enhanced availability and accessibility of quality, nutrient-rich and diversified food in targeted households, and improved consumption practices**						
1. Increase local food production among vulnerable households of nutrient dense rich foods for own consumption (all-year productive kitchen gardens, fruit trees)				X		8,9
2. Scale-up planting of nutritious trees and processing of their produce to mitigate the exisiting gaps in micronutrients with a focus on Spirulina and moringa among others				X		8
3. Increase coverage of vulnerable households supported to produce animal sourced foods for own consumption				X		8
4. Sustain Effective Food Consumption practices in vulnerable HH	X			X		5,8
5. Increase access to fortified foods to vulnerable households	X				X	5,10
7. Scale-up food fortification and bio-fortification efforts (protein iron rich beans, orange sweet potatoes, widely consumed food stuffs).					X	10
**Strategic Direction 5. Improved social protection systems for the poor and most vulnerable households**						
**Outcome 5. Increased access to and use of social protection services by targeted households to ensure adequate nutrition and access to IECD (Integrated Early Childhood**						
Output 5.2 Improved coverage, adequacy and appropriateness of social protection for reducing malnutrition, including nutrition-sensitive direct support, alternative income opportunities, food assistance and awareness raising on health, hygiene, and nutrition		X				6
**Strategic Direction 7. Strengthened community-based platforms to enhance demand for and use of effective frontline service delivery system of integrated high quality high**						
Outcome 7. Strengthened community-based platforms to enhance demand for and use of quality, integrated frontline ECD services						
4. Improve quality of measurements and reporting systems for child growth monitoring and promotion at community level	X					4
5. Strengthen community based nutrition programs including nutrition education counselling	X					3,5
a. Review and operationalize community-based screening and early identification systems of children at risk, including adoption of new methods (innovative Growth Monitoring and Promotion GMP at community level)	X					4
c. Scale up a package of prioritized community-based nutrition interventions to prevent and manage malnutrition in children less than 5 years, with a focus on those aged less than 2 for stunting and pregnant and lactating mothers	X					1,2,3,4
**National Food and Nutrition Policy Strategic Plan 2013-2018**						
**Key Outputs and Activities by Strategic Direction**	Health Services	Social Safety Nets	Educational Setting	Agriculture	Food Environments	10 Priority Candidates
**Strategic Direction 2. Prevent Chronic Malnutrition**						

**Output: Families and officials across Rwanda know and give value to the first 1000 days**						
Create national awareness on First 1000 Days around 1st 1000 days through national mass media, district media, organizational and non-formal channels	X					3
Effectively communicate the knowledge on the services and practices needed to protect children from stunting through a multichannel mix (mass, group and interpersonal) and formal education for families and caregivers throughout the country	X					1,2,3,4
**Output: Monthly 1st 1000 Days Community Based Food and Nutrition Programs with monthly sessions effectively balance anthropometric tracking with a wide range of**						
Organize and conduct monthly 1st 1000 days community based food and nutrition program activities including growth promotion activities with integrated early childhood (IEC) discussions with topics covering range of interventions to prevent stunting with participation from all pregnant women and families with children under 2 to participate	X					1,2,3,4
Design and implement at household level promotional activities including kitchen garden, small animal husbandry and production of staple foods				X		8
**Strategic Direction 3. Improving Household Food Security**						
**Output: Households increasingly diversify their food production**						
Establish village nurseries for fruit and agroforestry trees				X		8
Support development of homestead garden				X		8
Support development of small livestock				X		8
Establish model for nutrition gardens at village level				X		8
Provide food to very vulnerable groups (community kitchen programs)		X		X		6,8
**Output: Improve nutrition-related agricultural knowledge/practices of households**						
Prepare in Kinyarwanda "Book of nutritious local recipes" and disseminate locally	X			X		5,8
Contribute to updates of 1st 1000 Days Campaign	X			X		1,3,8
**Output: Income generating capacities of food and nutrition insecure households support**						
Provide technical and financial assistance to vulnerable households in greenhouse farming				X		8
Provide technical and financial assistance to vulnerable households in commercial vegetable and fruit production				X		8
**Output: Availability, affordability and quality of nutritious food improved**						
Provide extension and input support to producers of fortified beans and maize seeds and sweet potato vine				X	X	8,10
Implement national campaign for planting and consuming biofortified foods				X	X	8,10
Publicize the benefits of milk consumption for children	X			X		5,8
Support entrepreneurs to develop innovative milk products and packaging		X		X	X	6,9,10
**Output: Increased access to and use of appropriate food for most vulnerable households**						
Households of Ubudehe categories 1 and 2 targeted for nutritious complementary food during 1st 1000 days periods	X	X				3,6
Households of Ubudehe categories 1 and 2 targeted for direct cash transfers during 1st 1000 Days periods		X				6
Small livestock and seeds to Ubudehe categories 1 and 2 during their 1st 1000 Days periods		X		X		6,8
**Strategic Direction 4. Prevention and management of all forms of malnutritoin**						

**Output: Continued and strengthened activities to promote lowering of acute malnutrition**						
Build capacity for active identification and management of acute malnutrition through health facilities, routine and annual screening, referral and reporting based on growth monitoring and promotion in the 1st 1000 days campaign	X					4
Build capacity of health care providers at health facilities and CHWs on management of severe acute malnutrition through trainings and supportive supervision	X					4
**Output: Maternal Infant and Young Child Nutrition (MIYCN) effectively promoted**						
Reinforce optimal MIYCN through antenatal care visits, during health facility birth stays and throughout first two years by CHWs and health care providers at all levels and 1st 1000 Days campaign	X					1,3
**Output: Improve micronutrient nutrition**						
Conduct gap analysis on Fe+FA supplementation of pregnant women	X					1,5
Improve Fe+FA supplementation coverage and compliance for pregnant women and link to 1st 1000 days campaign	X					1,5
Sustain vitamin A supplementation in children > 5 and lactating women	X					5
Implement nationally new strategies to reach children 6-24 months of age with sufficient iron (in-home fortification using MNP and commercially fortified complementary cereals)	X					3,5
Promote and monitor implementation of 2013 regulation requiring staples foods fortification					X	10
Promote wide and rapid adoption and use of appropriate biofortified crops					X	10
Promote growing and use of mushrooms and highly nutritious crops				X	X	8,9,10
**Output: Prevention of obesity strategy developed and alliances formed to address growing prevalence of overnutrition and nutrition-related non-communicable diseases**						
Develop national strategy for obesity prevention	X	X			X	5,6,10
Create "Rwanda Alliance Against Obesity" with government, NGOs, RNS, civil society, private sector	X	X			X	5,6,10
Develop Rwanda Dietary Guidelines with adaptations for groups with different nutritional requirements including recommendations for physical activity	X					5
**Strategic Direction 5: Improving food and nutrition in schools**						
**Output: Food and nutrition education has been substantially expanded throughout school curriculum and extra-curricular activities**						
Conduct curriculum gap analysis focused on key elements of food and nutrition and life skills including 1st 1000 days			X			7
Develop curricular and extracurricular age appropriate leaning modules and activities expand food and nutrition learning components (urban vs. rural settings to be considered)			X			7
Produce and disseminate learning materials along with appropriate in service training of teachers on new learning modules/activities			X			7
Incorporate food and nutrition learning package into pre-.service training of teachers			X			7
Implement school gardens and “grow areas” as pedagogical tools for learning teaching about food and nutrition			X	X		7,8

Link school teaching and activities on food and nutrition with the key concepts, services and practices of the 1st 1000 Days campaign and community based nutrition programmes			X			7
**Output: The implementation of school feeding program has been expanded countrywide**						
Expand the "one cup of milk per child" in collaboration with MINAGRI		X	X			6,7
Operationalize and Institutionalize home-grown school feeding program			X			7
Sustain and improve the secondary boarding school feeding model			X			7
**Output: The School Health Policy has been implemented**						
Develop program to screen preschool and school children for malnutrition	X		X			4,7
Provide micronutrient supplements (vitamin A) to children under 5 in preschool	X		X			5,7
Collaborate with MOH on national strategy to eliminate anemia target groups particularly girls in secondary school (weekly campaign Fe+FA)	X		X			5,7

National Social Behavior Change and Communication Strategy for Integrated Early Childhood Development, Nutrition and WASH 2018-2024

**Priority Behaviors to Promote**	Health Services	Social Safety Nets	Educational Setting	Agriculture	Food Environments	10 Priority Candidates
**Adult Nutrition**						
Increase awareness that women of reproductive age, particularly pregnant and lactating women should eat meals four times each day that contain foods from at least four food groups out of seven food groups (demand).	X					1
Address taboos related to food and promote correct knowledge	X					5
Address food insecurity and rising price of nutritious food (access and resilience).		X		X		6,9
Promote reduced consumption of energy-dense food high in saturated fats and sugars, and increase physical activities among those who are obese.	X	X			X	5,6,10
**Infant and Young Child Nutrition**						
Promote good infant and young child feeding practices with a focus on quality of food as opposed to only the quantity of food; address resource allocation and prioritize nutritious food for children.	X					3
Promote optimal breastfeeding and early initiation of breastfeeding (within 30 min after delivery) and emphasis on colostrum.	X					2
Encourage adequate and timely complementary feeding to young children between 6 and 23 months.	X					3
Educate about minimum meal frequency; Minimum dietary diversity; adequate amount of food and consistency, active/responsive feeding, exclusive breastfeeding (not giving any other foods or liquids to infants besides breastmilk in the first 6 months of life).	X					2,3
**Health seeking behavior**						
Raise awareness to parents and caregivers to responsive child feeding practices	X					3
Promote regular attendance to the monthly growth monitoring and promotion sessions to assess the growth of the child.	X					3,4
Educate mothers and caregivers of children under 5 about the importance of complying with the instruction on how to give micronutrient powders to eligible children.	X					5
Promote full attendance at ANC/PNC visits	X					1

National School Health Strategic Plan 2014						
**Activities by Strategic Objectives**	Health Services	Social Safety Nets	Educational Setting	Agriculture		10 Priority Candidates
**Strategic Objective 5. Promotion of School Nutrition**						
Operationalize home-grown school feeding program (HGSFP) at all schools (pre-primary, primary, and secondary schools)			X			7
Continue other school feeding interventions (One cup of milk per child, secondary school feeding program)			X			7
Supplementation of micronutrients bi-annually (vitamin A) in schools	X		X			5,7
Promotion of nutrition education through school gardens			X	X		7,8
**Strategic Objective 6. Promotion of Physical Education**						
Strengthening of physical education and sports curriculum in schools (train PE/sports teachers in life skills and involve PTAs/local communities in implementation)			X			7
Promotion of sports activities to raise awareness (campaigns)			X			7
**National Strategy and Costed Plan for the Prevention and Control of Non-Communicable Diseases in Rwanda 2020-2025**						
**Strategic Actions by Strategic Objective and Priority Areas**	Health Services	Social Safety Nets	Educational Setting	Agriculture	Food Environments	10 Priority Candidates
**Strategic Objective 1. NCD Prevention through health promotion and reduction of risk factors**						
**Priority Area 1. Awareness-raising/education to reduce exposure to modifiable NCD risk factors**						
Establish a school health program for NCDs including injuries and disabilities increasing awareness and preventions as part of primary and secondary school curriculums	X		X			5,7
Promote community awareness of prevention of NCD risk factors	X					5
Empower districts to run NCD prevention and education programs	X					5
Develop and disseminate NCD prevention communication material targeting general population. These should focus on balanced diets, reducing alcohol consumption, promoting physical actvities and reducing tobacco addiction	X					5
Develop and integrate NCD-related education and awareness messages into package of service provided by all levels of health care and community health workers	X					3
**Priority Area 3. Establish and strengthen the implementation of policies and regulations addressing NCD risk factors**						
Establish regulations or policies related to healthy diets (e.g. trade of trans-fats, sugary beverages, processed food and sodium)		X			X	6,10
Conduct public awareness campaigns, including mass and social media, to inform and engage consumers about healthy diets based on locally available food and drink	X			X	X	5,9,10
Promote user-friendly or easy-to-understand food labelling on processed food packaging and translate international food labels into local languages		X			X	6,10
Reduce sugar consumption through increased taxation on sugar-sweetened beverages		X			X	6,10
Raise community awareness of healthy diets, including salt consumption	X				X	5,10
Promote farming with controlled use of agrochemicals, ensuring crop diversity, and cultivating fruits and vegetables				X		8
Develop and disseminate guidelines for the general population on physical activity and sports					X	10

Create enabling environments for promoting physical activity (e.g. road design and recreational spaces)					X	10
Increase general awareness on physical activity and benefits					X	10
**Strategic Objective 2. Strengthening health systems for quality NCD early detection, care and treatment at all levels**						
**Priority Area 1. Human resources development for NCD early detection, care and treatment**						
Train in-service health providers in early detection, diagnosis and management of NCDs including injuries and disabilities at all levels	X					3
Integrate national NCD prevention, early detection, care, and treatment guidelines into undergraduate pre-service training of the health workforce	X					3
**National Strategy for Transformation (NST1) 2017-2024**						
**Key Strategic Interventions by Priority Area and Pillar**	Health Services	Social Safety Nets	Educational Setting	Agriculture	Food Environments	10 Priority Candidates
**Social Transformation Pillar**						
**Priority Area 1. Promoting resilience and enhanging graduation from poverty and extreme poverty**						
Improve the management of the One Cow per Poor Family (Girinka) Program and other social programs run at the village level and support poor households to acquire small livestock		X		X		6,8
**Priority Area 2. Eradicating Malnutrition**						
Ensure and sustain food security by distributing food and vitamin supplements using Fortified Blended Food (FBF) and one cup of milk per child	X	X				5,6
Promote at village level the 1000 days of good nutrition and antenatal care by sensitizing households on good nutrition and hygiene practices through ECDs, health centers, and faith based campaigns	X					1,3,5
**Priority Area 3. Enhancing demographic dividend through ensuring access to quality health for all**						
Strengthen disease prevention awareness and reduce communicable and non-communicable diseases (NCDs) through community mobilization to prevent diseases through maintaining hygiene, promoting physical exercises for all age groups, regular health checkups and scaling up screening services in communities and health facilities	X					4
**National Social Protection Sector Strategic Plan (SP-SSP) 2018-2024**						
**Priority Actions by Outcome**	Health Services	Social Safety Nets	Educational Setting	Agriculture	Food Environments	10 Priority Candidates
**Outcome 2. Strengthened contribution of social protection to reducing malnutrition**						
Nutrition-sensitive direct income support provided to extremely poor mothers and infants		X				6
Develop guidelines delivery of sensitization on nutrition to ePW HBCC beneficiaries		X				6
Design behavioral change communication strategy (BCCS) to accompany VUP implementation		X				6
**Rubavu District Plan to Eliminate Malnutrition (DPEM) 2022-2023**						
**Activities by Output and Strategy**	Health Services	Social Safety Nets	Educational Setting	Agriculture	Food Environments	10 Priority Candidates
**Strategy 2. Prevent stunting in children under two years of age**						
**Output 1. Ensure community awareness and promote ECDs to prevent stunting among children under 2 years**						

Conduct nutrition and breastfeeding weeks	X					2,3
Coordinate Maternal Child and Community Health week	X					3
Conduct behavior change positive preventing sessions through home visitations	X					3
**Output 2. Provide nutrition sensitive direct support**						
Pay of beneficiaries (pregnant women and U2 children in Category 1) with NSDS (Nutrition-Sensitive Direct Support)		X				6
Conduct mobilization at community level on importance of 4 ANC standard visits by community health workers	X					1
Conduct screening of U2 children using Length Mat (stunting) in the community	X					4
Conduct screening of U5 children using MUAC (wasting) in the community	X					4
**Strategy 3. Strengthen, expand, and promote services and practices that result in household food security year round**						
**Output 1. Households increasingly diversity their food production**						
Distribute diversified seeds to ECD center				X		8
**Strategy 4. Prevent and manage all forms of malnutrition**						
**Output 1. Continued and strengthened activities to reduce acute malnutrition**						
Establish kitchen gardens to home-based ECD				X		8
Screen pregnant women in all health centers	X					1
**Output 2. Maternal Infant and Young Child Nutrition effectively promoted through providing holistic health and nutritional commodities**						
Provide nutrition support (milk to U5 children with malnutrition)		X				6
Provide FBF (fortified blended food) to children aged 6-23 months (Category 1 and 2)	X	X				5,6
Provide folic acid and iron supplements to pregnant women	X	X				1,5,6
Provide nutritional supplements to children	X					5
**Strategy 5. Strengthen nutrition education in schools and higher learning institutions through curricular and extracurricular activities**						
**Output 1. Food and nutrition education has been substantially expanded throughout school activities**						
Conduct monitoring of the availability and sustainability of school gardens				X		8

Rusizi District Plan to Eliminate Malnutrition (DPEM) 2022-2023

Activities by Output and Strategy	Health Services	Social Safety Nets	Educational Setting	Agriculture	Food Environments	10 Priority Candidates
**Strategic Direction 1. Children healthy and able to thrive**						
**Output 1: Maternal, Infant and Young Child Nutrition (MIYCN) effectively promoted through providing holistic health and nutritional commodities**						
Provide FBF to children under 2 years (Categorie 1&2)	X	X				5,6
provide Iron and Folic Acid to pregnant women	X	X				5,6
Provide FBF to pregnant and lactating women in Categories 1 and 2	X	X				1,5,6
Provide nutritional supplement to children (MNP, Vit A, RTUF, Ongera, etc.)	X	X				5,6
Screen children under 5 years for all forms of malnutrition with MUAC, height, length, weight, length mat	X					4
Transportation of FBF to additional sites	X	X				5,6
Conduct monthly growth monitoring of childrens under 5	X					4
Monitor the screening of children using LENGTH MAT	X					4
**Strategic Direction 2. Young children learn and reach their development potential**						
**Output 1: Food and nutrition education has been substantially expanded throughout school and Health facilities; curriculum and extra-curricular activities**						
Monitor the availability and sustainability of school gardens in all schools			X			7

**Output 2. Maternal Infant and Young Child Nutrition effectively promoted through providing holistic health and nutritional commodities**						
Provide ECDs with food and Porridge and basic learning materials	X		X			3,7
Organize and conduct the training of caregivers (Home and community based ECDs)	X					3
**Strategic Direction 3. Children are food secure and have a minimum standard of living**						
**Output 1. Local production of nutrient dense food crops among targeted HHs for own consumption through use of subsidiaries agricultural inputs (Fruit trees, Bio fortified**						
Construction of 2 model kitchen gardens in each village				X		8
Provide inputs (Seeds and Fertilisers)		X		X		6,8
Provide Fruit plants to vulnerable families		X		X		6,8
**Strategic Direction 6: Integrated frontline delivery**						
Organize and conduct radio talk shows on first 1000 Days of child life, balanced diet, breast feeding and hygiene practices	X	X				1,2,3,4,6
Growth screening and monitoring and basic food supplementation at village level	X					4,5
Organize and implement SBCC and cooking demonstrations at village level	X	X				3,6
Conduct home visits that include counseling on nutrition, WASH, and ECD Services as recorded on new checklist by CHWs	X					3
**Rwanda Agriculture and Animal Resources Development Board (RAB) Strategic Plan 2020**						
**Strategic Objectives by Outcome**	Health Services	Social Safety Nets	Educational Setting	Agriculture	Food Environments	10 Priority Candidates
**Outcome 2. Increased productivity, nutritional value, and resilience through sustainable, diversified, and integrated crop, livestock, and fish production systems**						
Promotion of nutrition sensitive agriculture (chicken, pigs, cows distributed)				X		8
Increasing kitchen gardens and school gardens			X	X		7,8
Promoting production and consumption of highly nutritious fruits and vegetables				X		8
**Strategic Plan for the Transformation of Agriculture Phase III (PSTA IV) 2018-2024**						
**Activities by Strategic Outcome**	Health Services	Social Safety Nets	Educational Setting	Agriculture	Food Environments	10 Priority Candidates
**1.3 Skills developed for agriculture value chain actors**						
**1.3.3 Women empowerment and skills development**						
Capacity building for developing skills and promoting increased involvement of women in agribusiness				X		8
Capacity building for developing leadership and management skills for women				X		8
**2.4 Nutrition sensitive agriculture**						
**2.4.1 Mainstreaming nutrition**						
Training of MINAGRI, RAB, and NAEB staff on NSA mainstreaming				X		8
Promote nutrition dense food e.g. iron fortified beans				X		8
Promote PPP models for food fortification				X	X	8,10
Nutrition education for farming households				X		8
**2.4.2 Upscaling of kitchen gardens program and home-grown school feeding program**						
Technical assistance for expanding kitchen garden program to promote more diversified diets at household level				X		8

Promote urban agriculture and agro-forestry (fruit trees, vegetables, small stock, fish ponds) program				X		8
Pilot nutrition campaigns in kitchen gardens				X		8
Subsidize kitchen gardens				X		8
Develop and expand school gardens			X	X		7,8
Source locally produced food for school feeding program			X	X		7,8
Contribute in the development of school curriculum with integrated nutrition education			X	X		7,8
**2.5 Mechanisms for increased resilience**						
**2.5.2 Asset building for vulnerable groups**						
Support vulnerable groups with chicken transfer, pig transfer		X		X		6,8
Girinka program		X		X		6,8
Asset transfer of other small-stock (goats, rabbits)		X		X		6,8
