# Supplementary material for: Evaluating Double-Duty Actions in Rwanda’s Secondary Cities
Source: Nutrients. 2024 Jun 23;16(13):1998. doi: 10.3390/nu16131998 (PMC11243673; doi:10.3390/nu16131998)
Supplement: Supplementary file 1 [file nutrients-16-01998-s001.zip › Supplementary File S2.pdf]

Appendix B. Interview Guide (English / Kinyarwanda)

<u>HEALTH SECTOR INTERVIEW GUIDE</u> / Umuyoboro w'ibazwa ku rwego rw'ubuzima

Health Sector Potential Interviewees:/**ABABAZWA B'INGENZI MU RWEGO RW'UBUZIMA**

- Directors of Health/**Umuyobozi ushinzwe ubuzima** (Rubavu and Rusizi Districts/**Akarere ka Rusizi na Rubavu**)
- ECD District Focal Points, Caregivers, Volunteers/ **Abayobozi b'ibanze bashinzwe amarerero y'incuke, abarezi n'abakorerabushake**
- Community Health Workers (CHWs)/**Abakozi bita k'ubuzima**
- Antenatal care providers/**Abita ku bagore batwite**
- Health-related NGO and FBO Representatives (ADEPE, African Evangelical Enterprise) **Imiryango itegamiye kuri leta yita k'ubuzima, abahagarariye imiryango ishingiye ku kwemera (ADEPE, Abashoramari mu ivugabutumwa Nyafurika**
- World Vision Rwanda members/**Abanyamuryango ba World vision Rwanda**

A. Stunting and wasting (undernutrition) in children under 5/Igwingira n'imirire mibi mu bana bari munsi y'imyaka 5

A1. *Assessing the Nutrition Problem/* ***Gusuzuma Ikibazo cy'imirire***
A1.1 In your experience, how common is stunting and/or wasting in children under 5? /**Mu bushishozi bwawe, Ubona hari igwingira n'imirire mibi mu bana bari munsi y'imyaka 5?**
If yes: Which children do you think are most affected? What do you think are the biggest contributing factors? **/Niba ari yego, ni abahe bana utekerezeko bagirwaho ingaruka nabyo? utekereza ko biterwa niki?**
If no: Please explain your view. Recent improvements or intervention successes? / **Niba ari oya, gerageza usobanure uko ubyumva. Igiheruka kugerwaho cyangwa igikorwa cyagezweho neza.**

A1.2 Have you seen recent improvement or worsening of these conditions? /**Hari impunduka nziza ubona cyangwa igituma biba bibi kurushaho muri iki kibazo?**
If yes: What do you think were the drivers of this change? /**Niba ari yego, utekereza ko ari iki gituma habaho izo mpinduka?**

A2. *Evaluating Interventions/* ***Gusuzuma ibikorwa***
A2.1 Are you aware of specific policies or programs that have been successful at targeting stunting and wasting (undernutrition) in children under 5? /**Waba uzi politiki cg gahunda yihariye yagezweho mu gukumira igwingira n'imirire mibi mu bana bari munsi y'imyaka 5?**
If yes: What has made these interventions successful? /**Niba ari yego, niki cyatumye ibyo bikorwa bigerwaho neza.?**

A2.2 Please review the relevant policies and programs identified through our desk review (below). Out of those listed, which interventions are you directly involved with? / **Gerageza usuzume politiki na gahunda bifitanye isano byagaragajwe binyuze mu isuzuma ryacu(hepfo), muri uru rutonde ni ubuhe buryo wagizemo uruhare?**
Prompt: Please describe your experience working in this intervention. / **Gerageza usobanure imikoranire yawe niyi gahunda?**
Prompt: What works the best? What works the least? / **Ni iyihe ikora neza cyane? Ni iyihe idakora neza?**
Prompt: What do you consider the biggest challenges**? / Ni iyihe mbongamizi nyamukuru wahuye nayo?**
Prompt: What do you think could be done to improve it? / **Ni iki utekerezako cyakorwa ngo bigende neza kurushaho?**

A2.3 Are there any other interventions targeting undernutrition in children under 5 that you're involved with that are not listed here? / **Haba hari indi gahunda cg igikorwa cyo gukumira igwingira n'imirire mibi mu bana bari munsi y'imyaka 5 wagizemo uruhare kitari mu byavuzwe hano?**

If yes: Please describe your experience working in this intervention. /**Niba ari yego, gerageza usobanure imikoranire yawe nicyo gikorwa**

**Relevant Interventions Identified by Desk Review/ibikorwa bifitanye isano byasuzumiwe hamwe**
Antenatal and post-natal care visits and counseling (nutrition, supplementation, breastfeeding, child feeding, positive parenting) **/ Gahunda yo kwita k'umugore utwite, gusura umugore ukimara kubyara no kumugira inama (kubijyanye n' imiririre myiza, imfashabere, konsa, kugaburira umwana, no kurera neza)**
Growth monitoring and screening of children under 5 at health centers **/ Gahunda yo gukurikirana no kugenzura imikuririre y'abana bari munsi y'imyaka 5 ku kigonderabuzima**
Vitamin A supplementation to lactating women/ **Guhabwa inyongera ya vitamin A ku bagore bonsa**
Iron and folic acid supplementation to pregnant women (ANC visits)/ **Guhabwa inyongera y' ubutare (feri) ku bagore batwite**
Iron supplementation to children 6-24 months / **Guhabwa inyongera y ubutare (feri) ku bana bari hagati y'amezi 6 na 24**
Distribution of fortified blended foods (Shisha Kibondo by Africa Improved Foods) to Ubudehe Category 1 and 2 households/**Gahunda yo gutanga Shisha Kibondo ku ngo ziri mu kiciro 1ni cya 2 cy'ubudehe.**
Nutrition education and counselling (ECDs, health centers, FBOs) /**Inyigisho kumirire myiza n'inama (ku Marerero y'incuke, ku bigonderabuzima, ibigo bishingiye ku kwemera)**
Home visits including counseling on nutrition, WASH, and ECD services/**Abantu basura ingo batanga inama ku mirire myiza, isuku na serivisi z'amarerero y'incuke.**
Food and porridge provided at ECDs**/ Gutanga ibiribwa n'igikoma ku marerero y'incuke.**
Awareness Campaigns: 1000 Days, Day of African Child, World Breastfeeding Week, Maternal and Child Health Week / **Ubukangurambaga ku'minsi igihumbi, umunsi w'umwana w'umunyafurika, icyumweru cyahariwe konsa, icyumweru cyahariwe ubuzima bw'umwana n'umubyeyi.**
In-home fortification: MNPs, complementary cereals / **Gukora imvange z'intungamubiri y'ibinyampeke mu rugo.**
Rwanda Alliance Against Obesity/Obesity prevention programs / **Ihuriro Nyarwanda mu gukumira umubyibuho ukabije**
NCD prevention and education programs /**Gahunda yo gukumira no kwigisha indwara zitandura**
One Egg per Child, Everyday/**Gahunda y'igi kuri buri mwana buri munsi**

B. Exclusive breastfeeding until 6 months/ Konsa umwana amezi 6 ntakindi umuvangiye

B1. *Assessing the Nutrition Problem/* ***Gusuzuma Ikibazo cy'imirire***

B1.1 In your experience, how long do women usually exclusively breastfeed after birth? **Mu bushishozi bwawe, ubona ababyeyi bakunda konsa abana igihe kingana gite ntakindi babavangiye Nyuma yo kubyara?**

Prompt: Which types of women are most/least likely to do so (e.g. young/old, urban/rural)? **Ni abahe bagore bakunda kubikora (urugero: abagore bakuze, bakiri bato, abagore bo mu cyaro cyangwa abagore bo mu muyji?**

B1.2 In your experience, are women aware of the benefits of exclusive breastfeeding for 6 months after birth? **/Mu bushishozi bwawe, ubona abagore bazi akamaro ko konsa umwana amezi 6 akivuka ntakindi amuvangiye?**

If yes: Where do most women receive this information? **/ Niba ari yego, ni hehe abagore bahabwa ayo makuru?**

B1.3 In your opinion, why do some women stop breastfeeding exclusively before 6 months? **/ Ku bwawe, ni ukubera iki abagore bahagarika konsa abana mbere igihe cy'amezi 6 ntakindi babavangiye?**

B1.4 Are you aware of any breastmilk substitutes sold or promoted in your city/district? **Waba hari imfashabere uzi zicuruzwa cyangwa zatejwe imbere mu mujyi/Akarere kawe?**

If yes: How popular do you think these substitutes are? **Niba ari yego, utekerezako izo mfashabere zizwi bingana iki?**

If yes: Do you think their availability contributes to lower rates of exclusive breastfeeding? **/Utekerezako kuboneka kwazo kugira uruhare mu kugabanuka ku mubare w'ababyeyi bonsa mu buryo budasanzwe?**

B2. *Evaluating Interventions/* ***Gusuzuma igikorwa***

B2.1 Are you aware of specific policies or programs that have been successful at improving breastfeeding practices? **/Waba hari politiki cyangwa gahunda yihariye uzi yagenze neza mu guteza imbere uburyo bwo konsa?**

If yes: What has made these interventions successful? **/ Niba ari yego, ni iki cyatumye iyo gahunda igenda neza?**

B2.2 Please review the relevant policies and programs identified through our desk review (below). Out of those listed, which interventions are you directly involved with? **Gerageza usuzume politiki na gahunda bifitanye isano byagaragajwe binyuze mu isuzuma ryacu(hepfo), muri uru rutonde ni ubuhe buryo wagizemo uruhare?**

Prompt: Please describe your experience working in this intervention. **Gerageza usobanure imikoranire yawe niyi gahunda?**

Prompt: What works the best? What works the least? **Ni iyihe ikora neza cyane? Ni iyihe idakora neza?**

Prompt: What do you consider the biggest challenges? **/Ni iki ufata nk' mbogamizi nyamukuru?**

Prompt: What do you think could be done to improve it? **/ Ni iki utekereza ko cyakorwa kugira ngo birusheho kugenda neza?**

B2.3 Are there any other interventions targeting exclusive breastfeeding that you're involved with that are not listed here? **/Haba hari indi gahunda urumo igamije konsa bidasanzwe (konsa ntakindi uvangiye umwana) urimo butavuzwe hano?**

If yes: Please describe your experience working in this intervention. **/ Niba ari yego, gerageza usobanure imikoranire yawe niyo gahunda**

**Relevant Interventions Identified by Desk Review/ ibikorwa bifitanye isano byasuzumiwe hamwe.**
Antenatal and post-natal care visits and counseling on breastfeeding/ **Gahunda yo kwita ku bagore batwite n'abamaze kubyara no kubaha inama zijyanye no konsa.**
ECD breastfeeding and positive parenting program for pregnant women/**Gahunda y'irerero ry'incuke,konsa no kurera neza ku bagore batwite.**
World Breastfeeding Week awareness campaign/**Gahunda y'ubukangurambaga ku cyumweru cya hariwe konsa ku isi.**

C. Overweight and obesity in women of reproductive age/Ibiro byinshi bikabije n'umubyibuho ukabije ku bagore bari mu myaka yo kubyara.

C1. *Assessing the Nutrition Problem/* ***Gusuzuma Ikibazo cy'imirirre***
C1.1 In your experience, how common is overweight and obesity among women? / **Mu bushishozi bwawe, umubyibuho ukabije n'ibiro byinshi bikabije bikunze kugaragara mu bagore?**
Prompt: Which types of women are most likely to be overweight? / **Ni abahe bagore bakunze kugira umubyibuho ukabije?**

C1.2 In your experience, are overweight women aware that they are overweight? / **Ukurikije uko ubibona, ubona abagore bafite umubyibuho ukabije bamenya ko bafite umubyibuho ukabije?**
Prompt: Are women in the community generally aware of the health risks of being overweight or obese? /**Abagore muri rusange bazi ingaruka k' ubuzima zo kugira umubyibuho ukabije cg Ibiro byinshi bikabije?**

C1.3 Have you noticed a difference in overweight prevalence between men and women? / **Waba hari itandukaniro wabonye hagati y'ubwiyongere bw'ibiro bukabije ku abagabo n'ubwiyongere bw' Ibiro bukabije ku bagore? /**
If yes: At what age do you begin to notice this difference? / **Ni kuyihe myaka utangira kubona iryo tandukaniro?**
Prompt: What do you think could be the reasons for this difference? **Utekereza ko ari iki gishobora kuba impamvu yiri tandukaniro?**

C1.4 Do you consider overweight and obesity among women to be a problem? /**Utekerezako Ibiro byinshi bikabije n'umubyibuho ukabije ari Ikibazo ku bagore?**
If yes: What do you think could be the reasons for this problem? / **Utekereza ko ari iki gishobora kuba Impamvu ziki kibazo?**

C2. *Evaluating Interventions/* ***Gusuzuma ibikorwa***
C2.1 Are you aware of specific policies or programs that have been successful at targeting overweight and obesity among women? /**Waba uzi politiki cg gahunda yagezweho neza mu kurwanya Ibiro byinshi n'umubyibuho ukabije ku bagore?**
If yes: What has made these interventions successful? / **Niba ari yego, ni iki cyatumye iyo gahunda igerwaho neza?**

C2.2 Please review the relevant policies and programs identified through our desk review (below). Out of those listed, which interventions are you directly involved with? / **Gerageza usuzume politiki na gahunda bifitanye isano byagaragajwe binyuze mu isuzuma ryacu(hepfo), muri uru rutonde ni ubuhe buryo wagizemo uruhare?**
Prompt: Please describe your experience working in this intervention. **Gerageza usobanure imikoranire yawe niyi gahunda?**
Prompt: What works the best? What works the least? **Ni iyihe ikora neza cyane? Ni iyihe idakora neza?**

Prompt: What do you consider the biggest challenges? **Ni iki ufata nk' imbogamizi nyamukuru?**
Prompt: What do you think could be done to improve it? / **Ni iki utekereza ko cyakorwa kugira ngo birusheho kugenda neza?**
C2.3 Are there any other interventions targeting overweight among women that you're involved with that are not listed here? **/Haba hari indi gahunda witabiriye igamije gukumira umubyibuho ukabije itari muzavuzwe hano?**
If yes: Please describe your experience working in this intervention. **/Niba ari yego, gerageza usobanure imikoranire yawe niyo gahunda.**

**Relevant Interventions Identified by Desk Review/ Ibikorwa byasuzumiwe hamwe**
Maternal and Child Health Week Awareness Campaign/**Gahunda y'icyumweru cyahariwe ubukangurambaga k'ubuzima bw'umwana n'umubyeyi.**
Rwanda Alliance Against Obesity/obesity prevention programs **/ Gahunda y'ihuriro Nyarwanda rigamije gukumira umubyibuho ukabije**

D. Anemia among women and children/ Ikibazo cy'amaraso make ku bana n'abagore

D1. *Assessing the Nutrition Problem/* ***Gusuzuma Ikibazo cy'imirire***
D1.1 In your experience, how common is anemia in the population? / **Mu bushishozi bwawe, ubona hari Ikibazo cy amaraso make mu baturage?**
Prompt: Which individuals or groups (e.g. age, gender) are most likely to be anemic? **Ni abahe bantu cg itsinda bakunze kwibasirwa (urugero: imyaka n'igitsina) n'ikibazo cy'amaraso make?**
D1.2 In your experience, are people in the community aware of the health risks of being anemic, especially while pregnant? / **Mu bushishozi bwawe, ubona abaturage bazi ingaruka zo kugira amaraso make cyane cyane ku mugore utwite?**
If yes: Where do most people receive this information? / **Niba ari yego, Nihehe abantu bahabwa ayo makuru?**

D2. *Evaluating Interventions/* ***Gusuzuma ibikorwa***
D2.1 Are you aware of specific policies or programs that have been successful at targeting anemia? **/ Waba uzi politiki cg gahunda yagezweho neza mu gukumira Ikibazo cy'amaraso make?**
If yes: What has made these interventions successful? / **Niba ari yego, ni iki cyatumye icyo gikorwa kigerwaho neza**

D2.2 Please review the relevant policies and programs identified through our desk review/ (below)/. Out of those listed, which interventions are you directly involved with? / **Gerageza usuzume politiki na gahunda bifitanye isano byagaragajwe binyuze mu isuzuma ryacu(hepfo), muri uru rutonde, ni ubuhe buryo wagizemo uruhare?**
Prompt: Please describe your experience working in this intervention. / **Gerageza usobanure imikoranire yawe niyi gahunda?**
Prompt: What works the best? What works the least? **Ni iyihe ikora neza cyane? Ni iyihe idakora neza?**
Prompt: What do you consider the biggest challenges? **Ni iyihe mbogamizi nyamukuru uhura nayo?**
Prompt: What do you think could be done to improve it? / **Utekereza ko ariki cyakorwa kugira ngo birusheho kugenda neza?**

D2.3 Are there any other interventions targeting anemia, especially among women and children under 5 years, that you're involved with that are not listed here? / **Haba hari ikindi gikorwa kigamije gukumira Ikibazo cy'amaraso macye cyane cyane ku bagore n'abana bari munsi y'imyaka 5 wakoranye nacyo kitari mu byavuzwe hano?**

If yes: Please describe your experience working in this intervention. / **Niba ari yego, gerageza usobanure imikoranire yawe nicyo gikorwa.**

**Relevant Interventions Identified by Desk Review/ibikorwa byasuzumiwe hamwe**
Antenatal and post-natal care visits and counseling (supplementation)/**igikorwa cyo kwita, gusura no kugira inama abagore batwite n'ababyaye**
Iron and folic acid supplementation to pregnant women (ANC visits)/ **igikorwa cyo gutanga inyongera y'ubutare (feri) na aside folike ku bagore batwite**
Iron supplementation to children 6-24 months/ **Igikorwa cyo guha inyongera y' ubutare (feri) ku bana bari hagati y'amezi 6-24**
Distribution of fortified blended foods (Shisha Kibondo) to Ubudehe Category 1 and 2 households (pregnant and lactating women, children 6-23 months)/ **Igikorwa cyo gutanga ifu y'igikoma ya Shisha kibondo ku miryango iri mukiciro cya 1 ni cya 2 cy'ubudehe (abagore batwite n'abonsa ,abana bari hagati y'amezi 6 na 23)**
In-home fortification: MNPs, complementary cereals/ **Igikorwa cyo kongera intungamubiri mu mvange y'ibinyampeke.**
Screening of pregnant women for anemia in all health centers/ **Igikorwa cyo kugenzura Ikibazo cy'amaraso macye ku bagore batwite mu bigonderabuzima byose.**

<u>EDUCATION SECTOR INTERVIEW GUIDE/</u>Umuyoboro w'ibazwa ku rwego rw'uburezi

Education Sector Potential Interviewees/**Ababazwa b'ingenzi mu rwego rw'uburezi:**

- School Feeding Program:/**Gahunda yo kugaburira abana ku ishuli**
- Director of Education, Joint Action Development Forum Officer, School Committee Members/ **Umuyobozi ushinzwe uburezi, Umukozi ushinzwe iterambere ry'ihururiro ry'ibikorwa, abanyamuryango b'ishuli**
- One Cup of Milk per Child Program: School Teacher Focal Point, Program Coordinator, Girinka Selection Officer/ **Gahunda y'igikombe cy'amata kuri buri mwana, umwarimu w'ishuri ry'ibanze, umuhuzabikorwa wa gahunda, Umukozi ushinzwe gutoranya abagenerwa girinka.**

D. Anemia among women and children/ Ikibazo cy'amaraso macye ku bagore n'abana

D1. *Assessing the Nutrition Problem/ **Gusuzuma Ikibazo cy' imirire***

D1.1 In your experience, how common is anemia in the population? /**Mu bushishozi bwawe, ubona hari ibura ry'amaraso mubaturage?**

Prompt: Which individuals or groups (age group) are most likely to be anemic? / **Ni irihe tsinda (imyaka n'itsinda) rikunze kwibasibwa nibura ry'amaraso?**

D1.2 In your experience, are people in the community aware of the health risks of being anemic, especially while pregnant? / **Mu bushishozi bwawe, ubona abantu bazi ingaruka zo kugira amaraso macye cyane cyane mu gihe cyo gutwita?**

If yes: Where do most people receive this information? /**Niba ari yego, nihehe abantu bahabwa ayo makuru?**

D2. *Evaluating Interventions/* ***Gusuzuma ibikorwa***

D2.1 Are you aware of specific school-related policies or programs that have been successful at targeting anemia in children? /**Waba uzi politiki cg gahunda yihariye yagezweho neza mu gukumira Ikibazo cy'amaraso macye mu bana**?

If yes: What has made these interventions successful? / **Niba ari yego niki cyatumye icyo gikorwa kigerwaho neza?**

D2.2 Please review the relevant policies and programs identified through our desk review (below). Out of those listed, which interventions are you directly involved with? **Gerageza usuzume politiki na gahunda bifitanye isano byagaragajwe binyuze mu isuzuma ryacu(hepfo), muri uru rutonde, ni ubuhe buryo wagizemo uruhare?**

Prompt: Please describe your experience working in this intervention / **Gerageza usobanure imikoranire yawe niyo gahunda.**

Prompt: What works the best? What works the least? **Ni iyihe gahunda ikora neza cyane? ni iyihe gahunda idakora neza?**

Prompt: What do you consider the biggest challenges? / **Niki ubona nk' imbongamizi nyamukuru?**

Prompt: What do you think could be done to improve it? / **Utekereza ko ari iki cyakorwa ngo birusheho kugenda neza?**

D2.3 Are there any other school-focused interventions targeting anemia in children that you're involved with that are not listed here? /**Haba hari kindi gikorwa cyibanda ku mashuri cyo gukumurira Ikibazo cy'amaraso macye mu bana mwakoranye kitari mu byavuzwe hano?**

If yes: Please describe your experience working in this intervention. / **Niba ari yego, gerageza usobanure imikoranire yanyu nicyo gikorwa**

**Relevant Interventions Identified by Desk Review /Ibikorwa byasuzumiwe hamwe**
Weekly campaign about Fe + FA to target anemia in girls in secondary schools/ **Icyumweru cyahariwe ubukangurambaga ku butare (feri) na aside folike mu gukumira Ikibazo cy' amaraso macye ku bana ba bakobwa mu mashuli yisumbuye.**

E. Dietary intake /Imirire

E1. *Assessing the Nutrition Problem/* ***Gusuzuma Ikibazo cy'imirire.***

E1.1 In your opinion, how do you think most community members define a healthy diet? /**Uko ubibona, utekereza ko abaturage benshi basobanura bate indyo yuzuye?**

Prompt: Which food groups are considered healthy or unhealthy? / **Ni irihe tsinda ry'ibibiribwa rifatwa nk'indyo yuzuye cg indyo ituzuye?**

Prompt: Where do you think most people get their information about healthy diets? / **Utekereza ko ari he abantu benshi bakura amakuru yerekeranye n'indyo yuzuye**?

Prompt: Do you notice a difference in perceptions between children and their parents? / H**ari itandukaniro ubona mu bitekerezo hagati y'abana n'ababyeyi babo?**

E1.2 What do you think the biggest dietary gaps are among children in the community? /**Utekerezako icyuho kinini mu mirere mu baturage kiri mu bana? /**

Prompt: Which foods or food groups are the least consumed or lacking in the diet? / **Ni ibihe biribwa cg itsinda ry'ibiribwa biribwa gacye cg bitaboneka mu ndyo?**

Prompt: Are any foods or food groups consumed in excess? / **Haba hari ibiribwa cg itsinda ry'ibiribwa biribwa ku kigero gikabije?**

E1.3 Which individuals or groups (e.g. age) do you think most often do not receive an adequate diet (sufficient quantity and/or diversity of foods) at home? / **Ni abahe bantu cg amatsinda (urugero; imyaka) utekerezako badakunze kubona indyo ihagije (ingano ihagije n'ubwoko butandukanya bw'ibiribwa)**
*Prompt***:** Why do you think these groups in particular do not receive an adequate diet? / **Utekerezako ari kubera iki ayo matsinda by'umwihariko atabona indyo ihagije?**

E2. *Evaluating Interventions*/ ***Gusuzuma ibikorwa***
E2.1 Are you aware of specific school-related policies or programs that have been successful at improving diets and dietary habits in children? / **Waba uzi politiki cg gahunda yihariye mu mashuli yagezweho neza mu gutezimbere indyo n'imirire ku bana?**
If yes: What has made these interventions successful? / **Niki cyatumye ibyo bikorwa bigenda neza?**

E2.2 Please review the relevant policies and programs identified through our desk review (below). Out of those listed, which interventions are you directly involved with? / **Gerageza usuzume politiki na gahunda bifitanye isano byagaragajwe binyuze mu isuzuma ryacu(hepfo), muri uru rutonde ni ubuhe buryo wagizemo uruhare?**
Prompt: Please describe your experience working in this intervention. / **Gerageza usobanure imikoranire yawe niyi gahunda**
Prompt: What works the best? What works the least? / **Ni iyihe ikora neza? Ni iyihe ikora gake?**
Prompt: What do you consider the biggest challenges? / **Niki ufata nk'inkombogamizi nyamukuru?**
Prompt: What do you think could be done to improve it? / **Utekerezako ari iki cyakorwa ngo bigende neza kurushaho?**

E2.3 Are there any other school-focused interventions targeting diets or dietary habits in children that you're involved with that are not listed here? **/ Hari indi gahunda yibanda ku mashuli mukwita ku biribwa n'imirire mu bana itavuzwe hano ugiramo uruhare?**
If yes: Please describe your experience working in this intervention. / **Niba ari yego, gerageza usobanure imirikoranire yawe niyi gahunda.**

**Relevant Interventions Identified by Desk Review/ Ibikorwa Byasuzumiwe hamwe**
School feeding program/ **Gahunda yo kugabura ku mashuli**
One Cup of Milk per Child program / **Igikombe cy'amata kuri buri mwana**.
School menu development to ensure nutritious meals for different age groups /**Gutegura urutonde rw'ibiribwa ku ishuli mu rwego rwo kunoza indyo yuzuye mu matsinda y'imyaka itandukanye**
School gardens and farming educational programs / **Gahunda y'ishuli y'karima k'igikoni no kwigisha ubuhinzi.**
School garden production used for school feeding / **Gukoresha akarima k'igikoni mu kugabura ku ishuli**
School feeding linked to local procurement (locally produced food) **/ Gahunda yo kugabura ku ishuli ihuzwa n' amasoko ya hafi (ibiribwa bihingwa hafi)**
Food and nutrition education expanded in curriculum and extracurriculars / **Inyigisho zidasanzwe zagutse ku biribwa n'imirire**
Parent involvement in school feeding and gardening programs / **Uruhare rw'ababyeyi muri gahunda yo kugabura no gutunganya akarima k'igikoni ku ishuli.**
Vitamin A supplementation to children under 5 in preschools / **Inyongera ya vitamin A ihabwa abana bari munsi**

**y'imyaka 5 bo mu kiburamwaka**
Inkongoro y'Umwana (children from poor households receive free milk at school / **abana bavuka mu miryango itishoboye bahabwa amata nta kiguzi ku ishuli**) /

<u>**SOCIAL PROTECTION SECTOR INTERVIEW GUIDE /**</u> **Umuyobora w'ibazwa ku rwego rurengera imiyoborere**

Social Protection Sector Potential Interviewees: **Ababazwa bingenzi mu rwego rurerengera imiyoborere.**

- Nutrition Sensitive Direct Support Program (NSDS) members / **Abagize gahunda yo gufasha abagirwaho ingaruka n'imirire**
- Shisha Kibondo ECD District Focal Point/ **Abahagarariye amarerero y'incuke mu turere**.
- National Women's Council, Executive Committee at District level/**Abagize komite nyobozi y'urwego rw'igihugu rw'abagore mu Karere.**
- Faith-based community volunteers in Rubavu and Rusizi / **Abakorerabushake bashingiye ku kwemera muri Rubavu na Rusizi**
- SUN Civil Society Alliance members in Rubavu and Rusizi / **Abagize ihuriro rya SUN CIVIL SOCIETY muri Rubavu na Rusizi**

A. Stunting and wasting (undernutrition) in children under 5 /Igwingira n'imirire mibi ku bana bari munsi y'imyaka 5

A1. *Assessing the Nutrition Problem/* ***Gusuzuma Ikibazo cy'imirire***
A1.1 In your experience, how common is stunting and/or wasting in children under 5? /**Mu bushishozi bwawe, hari igwingira n'imirire mibi mu bana bari munsi y'imyaka 5?**
If yes: Which children do you think are most affected? What do you think are the biggest contributing factors? / **Niba ari yego, ni abahe bana bigiraho ingaruka cyane? Utekereza ko ari iyihe mpamvu nyamukuru ibitera**?
If no: Please explain your view. Recent improvements or intervention successes? / **Niba ari oya, Gerageza usobanure uko ubibona. ibikorwa biheruka kugerwaho?**

A1.2 Have you seen recent improvement or worsening of these conditions? **Waba hari impinduka nziza uheruka kubona cg igutuma biba bibi kurushaho?**
If yes: What do you think were the drivers of this change? **Niba ari yego, utekerezako ari ki gitera izi mpinduka? /**

A2. *Evaluating Interventions/****Gusuzuma ibikorwa***
A2.1 Are you aware of specific policies or programs that have been successful at targeting stunting and wasting (undernutrition) in children under 5? **Waba uzi politiki cg gahunda yagezweho mugukumira igwingira n'imirire mibi ku bana bari munsi y'imyaka 5? /**
If yes: **What has made these interventions successful? Niba ari yego, ni iki cyatumye izo gahunda zigwerwaho neza?**

A2.2 Please review the relevant policies and programs identified through our desk review (below). Out of those listed, which interventions are you directly involved with? **Gerageza usuzume politiki na gahunda bifitanye isano byagaragajwe binyuze mu isuzuma ryacu(hepfo), muri uru rutonde ni ubuhe buryo wagizemo uruhare?**
Prompt: Please describe your experience working in this intervention. **Gerageza usobanure imikoranire yawe niyi gahunda**
Prompt: What works the best? What works the least? **Ni iyihe gahunda ikora neza cyane? ni iyihe gahunda idakora neza?**
Prompt: What do you consider the biggest challenges? **Niki ubona nk'inkombogamizi ikomeye?**

Prompt: What do you think could be done to improve it? **Utekerezako ari iki cyakorwa ngo bigende neza kurushaho?**

A2.3 Are there any other interventions targeting undernutrition in children under 5 that you're involved with that are not listed here? / **Haba indi gahunda igamije gukumira imirire mibi mu bana bari munsi y'imyaka 5 wagizemo urahare itari muzo twavuze hano?**
If yes: Please describe your experience working in this intervention. / **Niba ari yego, gerageza usobanure imikoranire yawe niyi gahunda.**

**Relevant Interventions Identified by Desk Review/ Ibikorwa byasuzumiwe hamwe**
Co-responsibility cash transfers during first 1000 days to Ubudehe 1 and 2 / **Gufatanya kohereza amafaranga mu minsi 1000 ya mbere mu cyiciro 1 ni cya 2 cy'ubudehe**
Distribution of fortified blended foods (Shisha Kibondo) to Ubudehe Category 1 and 2 households / **Gutanga imvange y'igikoma (shisha kibondo) ku miryango iri mu kiciro cya 1 ni cya 2 cy'ubudehe**.
Food assistance through community kitchen garden programs / **Inyunganizi ku biribwa bunyuze muri gahunda y'akarima k'igikoni mu baturage.**
Small livestock and seed distribution programs for Ubudehe 1 and 2 / **Gahunda yo gutanga imbuto n'ubworozi bw'amatungo buciriritse ku bari mu kiciro cya 1 ni cya 2 cy'ubudehe**

C. Overweight and obesity among women of reproductive age / Ibiro byinshi bikabije n'umubyibuho ukabije ku bagore bari mu myaka yo kubyara.

C1. *Assessing the Nutrition Problem/* ***Gusuzuma Ikibazo cy'imirire***
C1.1 In your experience, how common is overweight and obesity among women? / **Mu bushishozi bwawe, ubona umubyibuho ukabije ukunze kugaragara mu bagore?**
Prompt: Which types of women are most likely to be overweight? / **Ni abahe bagore bakunze kugira umubyibuho ukabije?**

C1.2 In your experience, are overweight women aware that they are overweight? / **Mu bushishozi bwawe ubona abagore bafite umubyibuho ukabije babimenya ko bafite umubyibuho ukabije?**
Prompt: Are women in the community generally aware of the health risks of being overweight or obese? **/Muri rusange mu baturage abagore bazi ingaruka zo kugira umubyibuho ukabije?**

C1.3 Have you noticed a difference in overweight prevalence between men and women**? /Waba warigeze ubona itandukaniro mukwiyongera k'umubyibuho ukabije hagati y'abagore n'abagabo?**
If yes: At what age do you begin to notice this difference? / **Niba ari yego, Ni kuyihe myaka utangira kubona iryo tandukaniro?**
Prompt: What do you think could be the reasons for this difference? / **Utekereza ko ari izihe mpamvu zitera iryo tandukniro?**

C1.4 Do you consider overweight and obesity among women to be a problem? / **Utekereza ko umubyibuho ukabije mu bagore ari ikibazo?**
If yes: What do you think could be the reasons for this problem? / **Utekereza ko ari izihe mpamvu zitera iki kibazo?**

C2. *Evaluating Interventions/* ***Gusuma ibikorwa***

C2.1 Are you aware of specific policies or programs that have been successful at targeting overweight and obesity among women? / **Waba uzi politiki cg gahunda yagezweho mu guhangana n'umubyibuho ukabije ku bagore?**
If yes: What has made these interventions successful**? / Niba ari yego, utekereza ko ari ki cyatumye bigerwaho?**

C2.2 Please review the relevant policies and programs identified through our desk review (below). Out of those listed, which interventions are you directly involved with? / **Gerageza usuzume politiki na gahunda bifitanye isano byagaragajwe binyuze mu isuzuma ryacu(hepfo), muri uru rutonde ni ubuhe buryo wagizemo uruhare?**
Prompt: Please describe your experience working in this intervention. **Gerageza usobanure imikoranire yawe niyi gahunda**
Prompt: What works the best? What works the least? **Ni iyihe gahunda ikora neza cyane? ni iyihe gahunda idakora neza?**
Prompt: What do you consider the biggest challenges? **Niki ubona nk'inkombogamizi ikomeye?**
Prompt: What do you think could be done to improve it? **Utekerezako ari iki cyakorwa ngo bigende neza kurushaho?**

C2.3 Are there any other interventions targeting overweight among women that you're involved with that are not listed here? **/ Haba hari indi gahund igamije gukumira umubyibuho ukabije ku bagore wagizemo uruhare ikaba itari muzavuzwe hano?**
If yes: Please describe your experience working in this intervention. **Gerageza usobanure imikoranire yawe niyi gahunda**

**Relevant Interventions Identified by Desk Review/ Ibikorwa byasuzumiwe hamwe**
Regulations and taxes on products high in trans-fat and sodium, sugary beverages, and processed food /**Amabwiriza n'imisoro ku biribwa bifite ibinure byinshi, sodiyumu, ibyo kunywa birimo isukari n'ibiribwa bitunganijwe mu nganda.**
Rwanda Alliance Against Obesity/obesity prevention programs / **Gahunda y'ihuriro Nyarwanda rigamije gukumira umubyibuho ukabije.**
User-friendly labeling and translated international food labels/ **Abakoresha ibirango bya gishuti n'ibirango mpuzamahanga by'ibiribwa byahinduwe**
Educational and behavior change campaigns on physical activity / **ubukangurambaga ku guhingura imyigire n'imyitwarire ku bikorwa ngororamubiri.**

<u>AGRICULTURE SECTOR INTERVIEW GUIDE/</u> Umuyoboro w'ibazwa mu rwego rw'ubuhnzi

Social Protection Sector Potential Interviewees:/ **Ababazwa bingenzi mu rwego rushinzwe imiyoborere.**
- One Acre Fund/Tubura Field Coordinators and Officers in Rubavu and Rusizi/ **Abahuzabikorwa n' abakozi ba Tubura muri Rusizi na Rubavu**
- Farmer Field Schools (FFS and Livestock FFS Facilitators in Rubavu and Rusizi/ **Abafashamyumvire mu ishuli ry'abahinzi n'aborozi muri Rubavu na Rusizi**
- Girinka Program Focal Point, program coordinator/**Umuhuzabikorwa wa gahunda ya Gira inka**
- School Gardening: School Committee members, Program coordinator / **Umuhuzabikorwa wa gahunda y'akarima k'igikoni ku ishuli n'abagize komite y'ishuli**
- World Vision Rwanda members/ **Abagize world vision yo mu Rwanda**

A. Stunting and wasting (undernutrition) in children under 5 years / Igwingira n'imirire mibi mu bana bari munsi y'imyaka 5

A1. *Assessing the Nutrition Problem* ***/ Gusuzuma Ikibazo cy'imirire***

A1.1 In your experience, how common is stunting and/or wasting in children under 5? / **Mu bushishozi bwawe, ubona igwingira n'imirire mibi bikunze kubaho ku bana bari munsi y'imyaka 5?**

If yes: Which children do you think are most affected? What do you think are the biggest contributing factors? / **Niba ari yego, ni abahe bana bikunze kugiraho ingaruka? Utekerezako biterwa niki?**

If no: Please explain your view. Recent improvements or intervention successes? **Niba ari oya, gerageza usobanure uko ubibona, haba hari igikorwa giheruka kugerwaho**

A1.2 Have you seen recent improvement or worsening of these conditions? / **Waba hari impinduka nziza uheruka kubona cg igutuma biba bibi kurushaho?**

If yes: What do you think were the drivers of this change? **Niba ari yego, utekerezako ari ki gitera izi mpinduka? /**

A2. *Evaluating Interventions* / ***Gusuzuma ibikorwa***

A2.1 Are you aware of specific agriculture-related policies or programs that have been successful at targeting stunting and wasting (undernutrition) in children under 5? **Waba uzi politiki cg gahunda yihariye mu buhinzi yagezweho mu gukumira igwingira n'imirire mibi ku bana bari munsi y'imyaka 5?**

If yes: What has made these interventions successful? **Niba ari yego, niki cyatumye iyo gahunda igerwaho neza?**

A2.2 Please review the relevant agricultural policies and programs identified through our desk review (below). Out of those listed, which are you directly involved with? / **Gerageza usuzume politiki na gahunda z'ubuhinzi bifitanye isano byagaragajwe binyuze mu isuzuma ryacu(hepfo), muri uru rutonde ni ubuhe buryo wagizemo uruhare**

Prompt: Please describe your experience working in this intervention. **Gerageza usobanure imikoranire yawe niyi gahunda**

Prompt: What works the best? What works the least? **Ni iyihe gahunda ikora neza cyane? ni iyihe gahunda idakora neza?**

Prompt: What do you consider the biggest challenges? **Niki ubona nk'inkombogamizi ikomeye?**

Prompt: What do you think could be done to improve it? **Utekerezako ari iki cyakorwa ngo bigende neza kurushaho?**

A2.3 Are there any other agriculture-related interventions targeting undernutrition in children under 5 that you're involved with that are not listed here? / **Haba hari indi gahunda yerekeranye n'ubuhinzi igamije gukumira imirire mibi ku bana bari munsi y'imyaka 5 wagizemo uruhare ikaba itavuzwe hano?**

If yes: Please describe your experience working in this intervention / **Gerageza usobanure imikoranire yawe niyi gahunda**

**Relevant Interventions Identified by Desk Review Ibikorwa byasuzumiwe hamwe /**
Promote research on biofortified crops and diversified animal products/ **Guteza imbere ubushakashatsi ku bihingwa bikomoka ku binyabuzima n'ibikomoka ku matungo atandukanye**
Girinka program (One cow per poor family) /**Gahunda ya Girinka (inka imwe kuri buri muryango ukennye)**

E. Dietary intake / Imirire

E1. *Assessing the Nutrition Problem* ***/ Gusuzuma Ikibazo cy'imiririre***

E1.1 In your opinion, how do you think most community members define a healthy diet? / **Ku bwawe, utekereza ko abaturage benshi basobanura bate indyo yuzuye**?
Prompt: Which food groups are considered healthy or unhealthy? / **Ni ayahe matsinda y' ibiribwa afatwa nkatanga ubuzima bwiza n'adatanga ubuzima bwiza?**
Prompt: Where do you think most people get their information about healthy diets? / **Utekereza ko abantu benshi bakura he amakuru ajyanye n'imirire myiza?**

E1.2 What do you think the biggest dietary gaps are in the community? **Niki utekereza ko ari icyuho kinini mu mirire mu baturage?**
Prompt: Which foods or food groups are the least consumed or lacking in the diet? **Ni ibihe biribwa cg itsinda ry'ibiribwa biribwa gake cg bitaboneka mu ndyo?**
Prompt: Are any foods or food groups consumed in excess? / **Haba hari ibiribwa cg itsinda ry' ibiribwa biribwa ku kigero gikabije?**

E1.3 Which individuals or groups (e.g. age) do you think most often do not receive an adequate diet (sufficient quantity and/or diversity of foods)? **/ Ni bahe bantu cg amatsinda (urugero: imyaka) utekereza ko badakunze kubona indyo ihagije (ingano ihagije, cg ubwoko butandukanya bw'ibiribwa)**
*Prompt***:** Why do you think these groups in particular do not receive an adequate diet? **Utekereza ko ari ukubera iki ayo matsinda atabona ibiribwa bihagije?**

E1.4 In your opinion, among farming households, which crops are most often produced? / **Ku bwawe, mu ngo z'abahinzi, ni ibihe bihingwa bikunze guhingwa?**
Prompt: Which crops are then mostly sold to the market and why? / **Ni ibihe bihingwa bikunze kugurishwa ku isoko, kubera iki?**
Prompt: Which crops are then mostly kept for at-home consumption and why? / **Ni ibihe bihingwa bijyanwa cyane kuribwa mu rugo, kubera iki?**

E2. *Evaluating Interventions*/ ***Gusuzuma ibikorwwa***
E2.1 Are you aware of specific agriculture-related policies or programs that have been successful at improving diets and dietary habits in the community? / **Waba uzi politiki cg gahunda ijjyanye n'ubuhinzi yagenze neza mu guteza imbere indyo n'imirire mu baturage?**
If yes: What has made these interventions successful? **Niba ari yego, niki cyatumye izo gahunda zigerwaho neza?**

E2.2 Please review the relevant policies and programs identified through our desk review (below). Out of those listed, which interventions are you directly involved with? **Gerageza usuzume politiki na gahunda bifitanye isano byagaragajwe binyuze mu isuzuma ryacu(hepfo), muri uru rutonde ni ubuhe buryo wagizemo uruhare?**
Prompt: Please describe your experience working in this intervention. **Gerageza usobanure imikoranire yawe niyi gahunda**
Prompt: What works the best? What works the least? **Ni iyihe gahunda ikora neza cyane? ni iyihe gahunda idakora neza?**
Prompt: What do you consider the biggest challenges? **Niki ubona nk'inkombogamizi ikomeye?**
Prompt: What do you think could be done to improve it? **Utekerezako ari iki cyakorwa ngo bigende neza kurushaho?**

E2.3 Are there any other agriculture-focused interventions targeting diets or dietary habits that you're involved with that are not listed here? **Haba hari ibindi bikorwa by'ubuhinzi byibanda ku ndyo n'imirire wagizemo uruhare bitari mubyavuzwe hano?**
If yes: Please describe your experience working in this intervention/ **Niba ari yego, gerageza usobanura imikoranire yawe n'ibi bikorwa.**

**Relevant Interventions Identified by Desk Review/ Ibikorwa byasuzumiwe hamwes**
Technical assistance for fruit trees and kitchen/homestead gardens in vulnerable households/ **Ubufasha muri tekiniki y'ibiti by'imbuto, akarima k'igikoni mu ngo zitishoboye.**
Girinka program (One cow per family)/ **Gahunda ya girinka (inka imwe kuri buri muryango)**
Vulnerable households supported to produce animal-source foods for own consumption through extension services/ **Ingo zitishoboye zifashwa gukora ibiryo bikomoka ku matungo binyuze muri serivisi zagutse**
Food fortification programs scale-up (iron-rich beans, maize, orange sweet potatoes) including extension and input support / **Gahunda yo kongera intungamubiri mu biribwa (ibishyimbo bikungahaye ku butare (feri), ibigoli, ibijumba bya karot**
Village nurseries for fruit agroforestry trees / **Ubuhumbikiro(pipiniyeri) bw'umudugudu bw'imbuto ziribwa n' izi biti bivangwa n'imyaka.**
School feeding linked to local procurement (locally produced food) / **Kugabura ku ishuli bihuzwa n'amasoko ya bugufi (ibiribwa bihingwa iwacu)**
Promoted growth of nutrient-dense crops (improved seeds, trainings) / **Gutezimbere gukura kw'ibihingwa bifite intungamubiri nyinshi (imbuto zivuguruye, amahugurwa)**
Targeted support to women for income-generating on- and off-farm activities / **Inkunga igenewe abagore mu kongera inyungu iva mu bikorwa by'ubuhinzi no mu bikorwa byo hanze y'ubuhinzi**
Chicken and/or pig transfer program for vulnerable households / **Gahunda yo gutanga inkoko cg ingurube ku ngo zikennye.**
Transfer programs of other small stock (goats, rabbits) / **Gahunda yo gutanga andi matungo magufi (ihene,inkwavu)**
Behavior change campaigns to encourage vegetable gardens and fruit trees / **Ubukangurambaga mu guhindura imyitwarire no gushishikariza kugira akarima k'imboga n'ibiti by'imbuto**.
Farmer Field Schools, Livestock Farmer Field Schools / **Ishuli ry'abahinzi, ishuli ry'abahinzi borzoi**

SUPPLEMENTARY MATERIAL/ Ibikoresho by'inyongera

Literature Review Summary (available to provide context)/ ***Ubuvanganzo bugaragaza ingingo z'incamake***

**Stunting/ igwingira**	
33.1% of children under 5 years in Rwanda are stunted **/ 33.1% by'abana bari munsi y'imyaka 5 mu Rwanda baragwingiye**	DHS/ ubushakashatsi Ku mibare n'ubuzima
In Rusizi and Rubavu stunting rates are 28.6% and 49.4% respectively/ **Muri Rusizi na Rubavu ikigero cy'igwingira kiri kuri 28.6% na 49.4%**	2021 NICE Baseline Survey/Ubushakashatsibw'ibanze bwa NICE 2021
Stunting is higher in rural areas and in food insecure households / **Igwingira rigaragara cyane mu bice by'icyaro no mungo zidafite ibyo kurya bihagije**.	DHS/ ubushakashatsi Ku mibare n'ubuzima
Stunting decreases with mother's education level and household wealth / **Igwingira rigabanukana n'ikigero cy'ubumenyi bw'ababyeyi ndetse n'ubukungu bw'urugo**.	DHS/ ubushakashatsi Ku mibare n'ubuzima
Leading hypothesis is stunting is linked to undernutrition in the first 1000 days of life / **Ibyavuye mu bushakashatsi bihuza igwingira n'imirire mibi mu minsi 1000 ya mbere y'ubuzima.**	UNICEF/ Umuryango W'abibumbye wita ku bana
**Wasting/ Imirire mibi**	
1.1% of children under 5 years in Rwanda are wasted / **1.1% by'abana bari munsi y'imyaka 5 mu Rwanda bafite imirire mibi**	DHS/ ubushakashatsi Ku mibare n'ubuzima /
In Rusizi and Rubavu wasting rates are 2.1% and 2.8 % respectively/ **Muri Rusizi na Rubavu ikigero cy'imiririre mibi ni 2.1% na 2.8%**	2021 NICE Baseline Survey / Ubushakashatsi bw'ibanze bwa NICE 2021
Wasting is higher in children with underweight mothers and highest in children 6-8 months / **Imirire mibi yiganje cyane mu bana bafite ababyeyi bafite umubyibuho ukabije ndetse no mu bana bari hagati y'amezi 6 -8**	DHS/ ubushakashatsi Ku mibare n'ubuzima
Leading hypothesis is wasting is linked to premature end of exclusive breastfeeding/ **Ubushakashatsi buhuza imiririre mibi no guhagarika konsa bidasanzwe imburagihe.**	UNICEF/ Umuryango W'abibumbye wita ku bana
**Exclusive breastfeeding/ Konsa umwana ntakindi ahabwa.**	
90.5% of women in Rwanda practice exclusive breastfeeding until 6 months / **90.5% by'abagore mu Rwanda bonsa abana ntakindi babaha kugeza ku mezi 6**	CFSVA / Isesengura Ryuzuye mu kwihaza mu biribwa
Exclusive breastfeeding until 6 months is lower in urban areas (86.7%) than in rural areas (91%)/ **Konsa ntakindi uha umwana kugeza ku mezi 6, biri hasi mu bice by'imijyi (86-7%) ugereranije n'ibice by'icyaro (91%)**	CFSVA/ Isesengura Ryuzuye mu kwihaza mu biribwa
Exclusive breastfeeding until 6 months is much lower in Rusizi (50%) and Rubavu (62.6%)	2021 NICE Baseline

than the national average / **Konsa mu gihe cya mezi 6 ntakindi umwana ahawe biri hasi muri Rusizi (50%) no muri Rubavu (62.2%) kurusha ikigereranyo cy'igihugu.**	Survey/ Ubushakashatsi bw'ibanze bwa NICE 2021
**Overweight and obesity in women of reproductive age / Ibiro byinshi bikabije n'Umubyibuho ukabije ku bagore bari mu myaka yo kubyara**	
26.3% of women 15-49 years in Rwanda are overweight and 5.8% are obese / **26.3 ku 100 by'abagore bari hagati y'imyaka 15 na 49 mu Rwanda bafite Ibiro byinshi bikabije na 5.8 ku 100 bafite umubyibuho ukabije.**	DHS/ ubushakashatsi Ku mibare n'ubuzima
Overweight in women 15-49 years in Rusizi is 32.9% and obesity is 7.9% / **Ibiro byinshi bikabije ku bagore bari hagati y' imyaka 15 na 49 bo muri Rusizi biri ku kigero cya 32.9 ku 100 nqho ubyibuho ukabije ukaba kuri 7.9 ku 100.**	2021 NICE Baseline survey Ubushakashatsi bw'ibanze bwa NICE 2021
Overweight in women 15-49 years in Rubavu is 36.4% and obesity is 7.9% / **Ibiro byinshi bikabije ku bagore bari hagati y' imyaka 15 na 49 bo muri Rubavu biri ku kigero cya 36.4ku 100 nqho ubyibuho ukabije ukaba kuri 7.9 ku 100.**	2021 NICE Baseline survey Ubushakashatsi bw'ibanze bwa NICE 2021
Prevalence of overweight and obesity in Rwandan women increases with age / **Umubare wa bagore babaNyarwanda bafite ibiro byinshi bikabije n'umubyibuho ukabije wiyongerana n'imyaka y'ubukure**.	DHS/ ubushakashatsi Ku mibare n'ubuzima
Overweight (42.2%) and obesity (22.3%) is higher among urban women compared to rural women (14% and 3.7% respectively) in Rwanda / **Mu Rwanda, ibiro byinshi bikabije (42.3%) n'umubyibuho ukabije (22.3%) biri hejuru ku bagore bo mu mujyi ugereranije n'abagore bo mu cyaro (14% na 3.7%)**	DHS/ ubushakashatsi Ku mibare n'ubuzima
Overweight and obesity in Rwandan women increases by education level **/ Ibiro byinshi Bikabije n'umubyibuho ukabije ku bagore baba Nyarwanda byiyongera uko ubumenyi Uko ikigero cy'ubumenyi cyiyongera**	DHS ubushakashatsi Ku mibare n'ubuzima /
Overweight and obesity in Rwandan women increases with household wealth / **Ibiro byinshi bikabije n'umubyiho ukabije ku bagore baba Nyarwanda byiyongera uko ubukungu bw'urugo bwiyongera**	DHS ubushakashatsi Ku mibare n'ubuzima /
**Anemia in women and children under 5/ Ikibazo cy'amaraso make ku bagore n'abana bari munsi y'imyaka 5**	
13% of women 15-49 years and 37% of children under 5 in Rwanda are anemic / **13 ku 100 by'abagore bari hagati y'imyaka 15 ba 49 mu Rwanda na 37 ku 100 by'abana bari munsi y'imyaka 5 bafite Ikibazo cy' amaraso macye.**	DHS ubushakashatsi Ku mibare n'ubuzima /
Anemia is highest among children 6-8 months (70.1%) and decreases with age / **Ikibazo cy'amaraso macye kiri hejuru mu bana bafite hagatiy'amazi 6-8(70-1 ku 100) kandi kigabanuka uko imyaka igabanuka**.	DHS ubushakashatsi Ku mibare n'ubuzima
Anemia is highest among women who are pregnant (24.5%) / **Ikibazo cy' amaraso macye kiri hejuru cyane ku bagore batwite (24.5 ku 100)**	DHS ubushakashatsi Ku mibare n'ubuzima /

Leading hypothesis is that anemia is linked to low maternal iron status and poor complementary feeding practices / **Ubushakashatsi bugaragaza ko kugira amaraso macye bifitanye i cy'ubutare (feri)kiri hasi ku babyeyi ndetse n'imirire ikennye.**	DHS ubushakashatsi Ku mibare n'ubuzima /
**Dietary Intake / Imirire**	
Less than 50% of children in Rwanda under 1 year are fed the minimum acceptable diet (MAD) for their age / **Hasi ya 50 ku 100 y'abana bari munsi y'umwaka 1 mu Rwanda bagaburirwa indyo yuzuye yemewe ku myaka yabo**	DHS ubushakashatsi Ku mibare n'ubuzima /
Children under 1 year in Rwanda living in rural areas are less likely to receive minimum acceptable diet (MAD) Than children in urban areas /**Abana bari munsi y'umwaka umwe mu Rwanda batuye mu nce z'icyaro ntibakunze kugaburirwa indyo yuzuye yemewe ugereranije n'abana batuye mu nce z'imijyi.**	DHS/ ubushakashatsi Ku mibare n'ubuzima
Children 6-8 months in Rwanda are the least likely to be fed adequate complementary foods / **Abana bari hagati y'amezi 6,8 mu Rwanda nibo badakunze kugaburirwa indyo yuzuzanya ihagije.**	CFSVA/ Isesengura Ryuzuye mu kwihaza mu biribwa
Proportion of children under 1 year in Rwanda fed MAD increases with mother's education /**Umubare w'abana bari munsi y'umwaka 1mu Rwanda bagaburirwa indyo yuzuye wiyongera uko ubumenyi bw'ababyeyi bwiyongera.**	DHS ubushakashatsi Ku mibare n'ubuzima /
Proportion of children under 1 year in Rwanda fed MAD increases with household wealth / **Umubare w'abana bari munsi y'umwaka 1mu Rwanda bagaburirwa indyo yuzuye wiyongera uko ubukungu bw'urugo bwiyongera.**	DHS ubushakashatsi Ku mibare n'ubuzima /
32% of Rwandan women 15-49 years meet minimum dietary diversity (MDD-W) / **32 ku 100 by'abagore baba Nyarwanda bari hagati y'imyaka 15 na 49 bafite imirire iboneye ntarengwa** .	CFSVA/ Isesengura Ryuzuye mu kwihaza mu biribwa
54% of Rwandan women 15-49 years in urban areas meet MDD-W compared to 28% in rural areas / **54 ku 100 by'abagore baba Nyarwanda bari hagati y'imyaka 15 na 49 batuye mu mujyi bafite imirire iboneye ntarengwa ugereranije na 28 ku 100 bo mu cyaro**	CFSVA/ Isesengura Ryuzuye mu kwihaza mu biribwa
Proportion of women meeting MDD-W in Rusizi is 39.3% and 26.7% in Rubavu / **Imibare y'abagore bafite imirere iboneye ntarengwa muri Rusizi ni 39.3 ku 100 na 26.7 ku 100 i Rubavu**	2021 NICE Baseline survey Ubushakashatsi bw'ibanze bwa NICE 2021
Women in Rwanda have low consumption of vitamin A rich food, meat, milk, eggs, and fruit /**Abagore bo mu Rwanda bafungura ku kigero cyo hasi ibiribwa bikungahaye kuri vitamin A, inyama, amata, amagi n'imbuto**	CFSVA/ Isesengura Ryuzuye mu kwihaza mu biribwa
Children in Rwanda have low consumption of eggs, flesh food, and dairy products / **Abana bo mu Rwanda bafungura ku kigero cyo hasi amagi, ibiryo byiza, n'ibikomoka ku mata**	CFSVA/ Isesengura Ryuzuye mu kwihaza mu biribwa
