# Supplementary material for: Evaluating Double-Duty Actions in Rwanda’s Secondary Cities
Source: Nutrients. 2024 Jun 23;16(13):1998. doi: 10.3390/nu16131998 (PMC11243673; doi:10.3390/nu16131998)
Supplement: Supplementary file 1 [file nutrients-16-01998-s001.zip › Supplementary File S3.pdf]

**Appendix C.** DDA program descriptions

| Program | Description |
|---|---|
| 1. Antenatal care (ANC) visits | Women are encouraged to attend eight ANC visits from the moment they know they are pregnant. At these visits they receive health screenings, vitamin supplements (iron and folic acid) and nutrition counselling. |
| 2. Awareness campaigns/Community mobilization sessions | Community gatherings are held once a week across the country during which local leaders make announcement about national programs (e.g. school feeding) and community interventions (e.g. small stock distribution). Occasionally, these gatherings include educational sessions about proper nutrition. |
| 3. Early Childhood Development (ECD) centers | These community centers provide services to children 3 to 6 years old to prevent delay in brain stimulation and to ensure healthy growth before primary school. Services include: education, nutrition, sanitation, and protection. ECDs also provide support to pregnant and breastfeeding women through education sessions. |
| 4. Exclusive breastfeeding promotion | Counselling about exclusive breastfeeding until 6 months and proper complementary feeding practices is given at ANC visits, ECDs, and community sessions with community health workers (CHWs). |
| 5. Farmer Field Schools (FFS) | This agricultural extension program recruits and trains facilitators in best agricultural practices to pass on to other farmers in their communities. FFS facilitators use small plots of land (demo plots) to demonstrate planting local , nutritious crops and using modern and sustainable farming techniques. |
| 6. Fruit trees | This campaign aims to mobilize every household in Rwanda to plant at least three different fruit trees to encourage fruit consumption in the home. The campaign also encourages schools and public offices to plant trees on their property to set an example in the community. |
| 7. Kitchen gardens | This program mobilizes households to use a small plot on their land or in their homes to plant local and nutritious vegetables reserved for home consumption. Public institutions, such as schools and district offices, must also have model kitchen gardens on their property where demonstrations can take place on how to initiate and maintain a kitchen garden. |
| 8. NCD prevention and physical activity promotion | At least one mass sports event is organized monthly in all districts followed by an education session on the importance of physical activity and proper nutrition to prevent overweight and obesity. |

| | |
|---|---|
| 9. Nutrition-Sensitive Direct Support (NSDS) and Shisha Kibondo | NSDS is a cash transfer scheme targeting poor households with pregnant women and/or children under 2 years to purchase nutritious foods and health services. Shisha Kibondo, a fortified blended food (FBF), is a maize-corn blend with vitamin/mineral premix given to women from vulnerable households who have a child identified as malnourished. |
| 10. School feeding program | The aim of this program is to ensure that every Rwandan child receives at least one healthy meal per day if they attend school. The average cost of a base meal for one child is 150 RWF. While the government subsidizes some of this cost per child (56 RWF), the child's parents are responsible for covering the rest. Parents unable to contribute money can choose to contribute food, materials, or small jobs instead. |
| 11. Small stock distribution | Distribution of small stock (pigs, poultry, sheep, goats) to vulnerable families is led by youth and women cooperatives. The goal is to increase access to manure and income among vulnerable families. The beneficiaries of these programs are selected by the community during community gatherings. |
| 12. Trainings (caregivers, teachers, CHWs) | Caregivers, teachers, and CHWs attend trainings on a variety of topics including nutrition and preparation of healthy diets that are organized by government or development partners and usually occur 1-3 times per year. |
