# Supplementary material for: Evaluating Double-Duty Actions in Rwanda’s Secondary Cities
Source: Nutrients. 2024 Jun 23;16(13):1998. doi: 10.3390/nu16131998 (PMC11243673; doi:10.3390/nu16131998)
Supplement: Supplementary file 1 [file nutrients-16-01998-s001.zip › Supplementary File S4.pdf]

Appendix D. Final SWOT Analysis Tables

D1. Antenatal Care Visits and Counselling

STRENGTHS	WEAKNESSES
**S1**. CHW education sessions ensure they are well trained<br>**S2**. Free iron and folic acid supplements provided to pregnant women<br>**S3**. Household visits by CHWs encourage attendance and ensure women are following advice<br>**S4**. Local leaders and para-socialsS encourage ANC visit attendance<br>**S5**. Shisha Kibondo provided at visits	**W1**. Some women don't attend sessions or only attend few<br>**W2**. No budget for providing incentives for attendance
**OPPORTUNITIES**	**THREATS**
**O1**. Increase recommended number of ANC visit from 4 to 8<br>**O2**. Provide incentives for attending ANC visits (e.g. soaps, umbrellas)<br>**O3**. Change micronutrient (Fe, FA) supplement pack to only last 1 month instead of 2<br>**O4**. CHWs conduct household visits to pregnant women on a monthly basis<br>**O5.** Provide Information Education Communication sessions at health centres (family planning, nutrition, maternal health)	**T1**. Superstition about showing pregnancy (women hide pregnancy out of fear)<br>**T2**. Mindset (misunderstanding that health centres are for sick people)<br>**T3**. High cost of healthy food and belief that nutritious food is only for wealthy people<br>**T4**. Lack of health insurance<br>**T5**. Women are working and have no time to attend ANC sessions<br>**T6**. Women have many children and cost of feeding them healthy meals is too high<br>**T7**. Women delay ANC visits to avoid having to pay for all sessions

D2. Awareness Campaigns/Community Mobilization Sessions

STRENGTHS	WEAKNESSES
**S1**. Knowledge of healthy diet has improved since NICE social marketing campaign (Neeza), especially due to brand recognition<br>**S2**. People are motivated to learn and apply changes when campaigns are well designed<br>**S3**. Collaboration with local government or leaders to run mobilization (e.g. church leaders)	**W1**. Low attendance at mobilization/education sessions especially among most vulnerable households<br>**W2**. Low male or father engagement

OPPORTUNITIES	THREATS
**O1**. Target awareness campaigns to places of work or homes<br>**O2**. Include memorable taglines, logos, images, etc. in campaigns<br>**O3**. Provide uniforms for CHWs leading education sessions<br>**O4**. Run radio programs and mobilization sessions to encourage men specifically to partake in nutrition interventions (men encouraging men)<br>**O5.** Use physical demonstrations at community gatherings (e.g. how to prepare a balanced diet, how to practice proper hygiene)	**T1**. No time to attend campaigns or education sessions in the community<br>**T2**. Lack of trust or respect for facilitators leading the education sessions<br>**T3**. Lack of capacity or confidence to implement lessons taught through mobilization

D3. Early Childhood Development (ECD) Centres

STRENGTHS	WEAKNESSES
**S1**. Parents see changes in children and are motivated to follow ECD model at home<br>**S2**. Home visits show that parents start implementing kitchen gardens after seeing them at ECDs<br>**S3**. Training of ECD caregivers improves quality of services and trainings	**W1**. Low parent monetary contribution<br>**W2**. Inconsistent and unequal support across ECDs (meals, materials, caregiver support)<br>**W3**. Not enough food at ECDs for all children<br>**W4**. Lack of fruits and animal products in meals provided at ECDs
**OPPORTUNITIES**	**THREATS**
**O1.** Option for parents to make non-monetary contributions (food; firewood and charcoal; cooking, cleaning, gardening services)<br>**O2**. Increased trainings for caregivers (ensuring continuous education)<br>**O3**. Increased parent involvement (e.g. parents form groups to raise money to improve ECDs)<br>**O4**. Income or incentives for caregivers (money is best incentive even if small amount)<br>**O5.** Close follow-up (home visits to monitor changes or adoption of practices)<br>**O6**. Provide incentives for caregivers to attend trainings	**T1**. Shared mindset that ECDs are a government program and responsibility<br>**T2**. High cost of healthy food at market (high price volatility)<br>**T3**. Lack of sufficient funding<br>**T4**. Caregivers not compensated or incentivized to attend trainings and many do not have the time<br>**T5.** Stock-outs (porridge stock-outs lead to drop-outs or ECD closures)<br>**T6.** Lack of long-term program sustainability without partner or government support<br>**T7**. Inconsistent government budget (leads to stock-outs)

**O7**. Introduce attractive new topics for different trainings based on what caregivers want	**T8**. Lack of knowledge (parents do not know or value proper ECD services)<br>**T9.** Parents do not have the time to attend ECD trainings or education sessions

D4. Exclusive Breastfeeding

STRENGTHS	WEAKNESSES
**S1**. Education materials (books) about 1000 days and proper breastfeeding<br>**S2**. Growth monitoring program is effective and complemented with breastfeeding education	**W1**. Some lactating mothers are malnourished and don't have enough breastmilk to feed<br>**W2**. Some poor mothers choose to leave baby at home to find work
**OPPORTUNITIES**	**THREATS**
**O1**. Provide Shisha Kibondo or FBF to malnourished pregnant or lactating women to help them improve nutrition status<br>**O2**. Encourage women to leave breast milk at home when going to work<br>**O3**. Designated spaces for breastfeeding (at workplaces, in public spaces)<br>**O4**. Education about importance of breastfeeding at community gatherings	**T1**. Lack of knowledge about breastfeeding<br>**T2**. Mothers have to work and are out of the house from early morning until evening<br>**T3**. Mothers lack of trust or confidence in letting others feed their babies for them

D5. Farmer Field Schools

STRENGTHS	WEAKNESSES
**S1.** SMS reminders for farmers (when to top dress, weed, harvest) work well when in-person follow-up is impossible<br>**S2.** Model plot hands-on demonstrations effective at engaging farmers<br>**S3**. Farmers reached through the program are motivated to learn new methods	**W1**. Animal products are the first thing farmers sell on the market (not kept for at-home consumption)<br>**W2**. Lack of feed industry in many districts results in high costs and long wait times to acquire feed<br>**W3.** Often there are not enough trained FFS facilitators to cover all farmers
**OPPORTUNITIES**	**THREATS**
**O1.** Improved quality of feed for small stock (alternative methods e.g. black soldier flies)<br>**O2.** Continuous education for farmers and follow-up from FFS facilitators	**T1.** Farmers prioritize selling production to buy land or other inputs rather than consuming it<br>**T2**. Farmers who don't receive follow-up become disincentivized to continue implementing best practices

**O3.** More frequent and recurring trainings for FFS facilitators on best practices and on facilitation	**T3.** Inputs and feed are expensive on the market (organic fertilizers especially)<br><br>**T4**. Shared mindset to value quantity of production over quality<br><br>**T5.** Belief that nutritious food is for wealthy people<br><br>**T6.** Small stock often fall ill (due to bad practices or poor quality of feed)

D6. Fruit trees

STRENGTHS	WEAKNESSES
**S1**. Government initiative to have 3 fruit trees per household has increased adoption significantly	**W1**. Low survival rate of fruit trees (animals destroy them, lack of proper care, etc.)
**OPPORTUNITIES**	**THREATS**
**O1**. Provide seeds or seedlings as small but long-term investment<br><br>**O2**. Invest in improving the quality of fruit tree seeds (e.g. modified seeds) with lower inputs and land needs<br><br>**O3**. Increase adoption of trees in kitchen and school gardens<br><br>**O4**. Increase mobilization about nutritional and economic benefits (diversified diet and income)<br><br>**O5**. Collaborate with local government and leaders (especially on modified seeds)<br><br>**O6**. Provide discounted seedlings over free fruit (higher success rate)	**T1**. Lack of land to plant trees (in homes and schools)<br><br>**T2**. Selling fruit on the market to buy other foods instead of feeding first to the family<br><br>**T3**. Lack of monitoring and follow-up (survival rate is low because no one is held accountable)<br><br>**T4**. Inputs are expensive (organic fertilizer especially)<br><br>**T5**. Disasters and shocks (Russia-Ukraine war, COVID, floods)

D7. Kitchen gardens

STRENGTHS	WEAKNESSES
**S1**. Model gardens at ECDs and in community serve as education opportunities to parents and incentivize them to start their own kitchen gardens<br><br>**S2**. People are motivated to adopt changes and keep food for families	**W1**. Low adoption in urban areas where families do not have sufficient space or capacity in their homes
**OPPORTUNITIES**	**THREATS**
**O1**. Increase education sessions about specific nutritious vegetables easy to grow in small spaces	**T1**. Many families discouraged by lack of land or capacity

**O2**. Increase education sessions and demonstrations about how to prepare meals with vegetables from garden<br>**O3**. Scale up school and ECD gardens<br>**O4**. Close follow-up at people's homes to monitor adoption and improvements<br>**O5**. Provide vegetable seeds to parents of children with SAM with education on how to grow and eat them<br>**O6**. Mobilization about the benefits of vegetables and fruits for at-home consumption<br>**O7.** Make free manure available for collection at public dumping sites for biological waste (Rusizi)	**T2**. Gender-based violence and family conflict prevent women from making changes or decisions in the home<br>**T3**. Urbanization results in smaller land per household<br>**T4**. Costs including bags for soil, manure, transportation<br>**T5**. Families prioritize selling produce from gardens instead of consuming<br>**T6**. Women don't have time to add more responsibilities to their home duties

D8. NCD prevention programs

STRENGTHS	WEAKNESSES
**S1**. Mass sport sessions organized every month followed by health education sessions about proper nutrition for NCD and obesity prevention (reduce oils, sugar, salt)<br>**S2**. Growing awareness of the risks of overweight and obesity<br>**S3**. Good participation in sports among youth (Rubavu especially)<br>**S4**. Health care practitioners at health centres educated and trained about NCD prevention	**W1**. Little awareness or acceptance of NCDs and obesity as a nutrition-related problem<br>**W2**. Low participation in sports among women<br>**W3**. No specific interventions exist to encourage women to exercise despite them being at the highest risk of overweight and obesity
**OPPORTUNITIES**	**THREATS**
**O1**. Encourage women to exercise and participate in sports<br>**O2**. Women leaders in sports (e.g. teachers at schools)<br>**O3**. Increase education and awareness about health risks of overweight and obesity and links to NCDs<br>**O4**. Hold physical competitions in schools and in the community	**T1.** Adults less motivated to participate in physical activity than youth<br>**T2**. Mindset that being overweight is a sign of wealth<br>**T3**. Women are ashamed or embarrassed to exercise in public<br>**T4**. Exercise activities and sports are predominantly run by and for men<br>**T5**. Women stop exercising and change diets after marriage and pregnancy<br>**T6**. Women have no time to participate in physical activity and do not see it as a priority

D9. NSDS and Shisha Kibondo

STRENGTHS	WEAKNESSES
**S1**. Stunting prevention measures taken before birth and continued into childhood	**W1**. Big families with many children rarely see effects of Shisha Kibondo as they are often distributing the Shisha Kibondo among all children in the household rather than only serving to the malnourished child
**OPPORTUNITIES**	**THREATS**
**O1**. Close monitoring through home visits to ensure Shisha Kibondo is properly prepared and consumed<br>**O2**. More holistic support to vulnerable family instead of to just one child<br>**O3**. Administer Shisha Kibondo outside the home, e.g. directly in health facilities or ECDs	**T1**. Provides contradictory messaging to family planning interventions<br>**T2**. Beneficiaries sell Shisha Kibondo rather than consuming it<br>**T3**. Beneficiaries dilute and share Shisha Kibondo among many children at home<br>**T4**. Forgotten age group (support lasts only until 2 years)

D10. School feeding program

STRENGTHS	WEAKNESSES
**S1**. Non-monetary contributions by parents can help subsidize school meals (e.g. small fish, peanuts, sweet potatoes)<br>**S2**. Parent meetings and mobilization increase awareness, motivation, and contributions<br>**S3**. Daily school meals have improved school attendance and focus in class	**W1**. Low parent contribution<br>**W2**. Lack of animal products or vegetables or fruits in the menu (always maize, beans, rice, potatoes, soya)
**OPPORTUNITIES**	**THREATS**
**O1**. Increase collaboration between parents and school to increase contribution and involvement<br>**O2**. Increase collaboration with local government or leaders to encourage parents<br>**O3**. Run trainings at schools for parents on starting and maintaining vegetable gardens<br>**O4**. Increased kitchen and eating materials to meet demand (e.g. "muvero", plates, utensils, cooking needs).	**T1**. Parents, especially those with many children, are unwilling or cannot afford to contribute to the program (contribution is per child)<br>**T2**. Mindset that school feeding is government's responsibility<br>**T3**. No specific room for eating (refectory)<br>**T4**. Lack of trust or respect for teachers or school feeding committee

	**T5**. Lack of kitchen materials (makes food preparation time consuming)

D11. Small stock distribution

STRENGTHS	WEAKNESSES
**S1**. Livestock to poor families (Girinka) has shown impressive improvements in nutrition<br>**S2**. Diversification in income<br>**S3.** Population decides who to prioritize for small stock	**W1**. Farmers don't replace livestock after they die or are sold<br>**W2**. Livestock are not well cared for or fed and fall sick
**OPPORTUNITIES**	**THREATS**
**O1**. Complement livestock (Girinka) program with small stock (chicken, birds, rabbits, goats) given their shorter reproduction rate, easier handling, and less expensive care<br>**O2**. Complement livestock (Girinka) program with fruit tree seedling program (agroforestry) to diversify income<br>**O3**. Close follow-up and monitoring to encourage farmers not to sell animals<br>**O4**. Select groups and farmers who have proven to be responsible to be recipients	**T1**. Many discouraged by lack of capacity (old, weak, physically disabled)<br>**T2.** Mindset that program is responsibility of the government or partners (unsustainable)<br>**T3**. Lack of land and shelter<br>**T4**. Farmers will sell production rather than keeping or reserving for home consumption<br>**T5**. Poor quality of feed or high cost of feed due to no local producer<br>**T6**. Insufficient veterinarians or inadequate veterinary services in rural areas

D12. Trainings (teachers, CHWs, caregivers)

STRENGTHS	WEAKNESSES
**S1**. Trainings provided to CHWs and ECD caregivers from various partners covering a range of nutrition topics (balanced diet, growing vegetables, 1000 days, etc.)<br>**S2**. CHWs also act as community facilitators and share learnings from trainings in community<br>**S3**. CHWs and caregivers are enthusiastic about trainings	**W1**. Trainings are too few and not repeated enough
**OPPORTUNITIES**	**THREATS**
**O1**. Introduce attractive new topics in different trainings (e.g. how to grow new nutritious vegetables like mushrooms)<br>**O2**. Have new faces giving trainings<br>**O3**. Provide incentives for caregivers and CHWs to attend trainings (bus tickets for transportation, free books or materials)	**T1**. Caregivers and CHWs not compensated or **incentivized** for attending trainings, many do not have the time<br>**T2**. Lack of follow-up or refresher trainings hinder long-term learning

**O4**. Provide more materials for use during and after trainings to improve long-term learning (e.g. 1000 Days book)<br>**O5**. Increase refresher trainings and continuous education	
