# Supplementary material for: Evaluating Double-Duty Actions in Rwanda’s Secondary Cities
Source: Nutrients. 2024 Jun 23;16(13):1998. doi: 10.3390/nu16131998 (PMC11243673; doi:10.3390/nu16131998)
Supplement: Supplementary file 1 [file nutrients-16-01998-s001.zip › Supplementary File S5.pdf]

Appendix E. Recommendations by Program

Recommendations by Program	SWOTs Addressed
**1. Antenatal Care Visits and Counseling**	
R1. Provide rewards for women who attend all 8 ANC visits	**O1, O2, W1, T2**
R2. Open egg or small fish retailer next to health centres with discounted prices for women who attend ANC visits where they can place orders to pick up at their next visit	**O1, O2, W1, W2, T3, T6**
R3. Incorporate family planning and mental health counselling at visits	**S1, O5, T1, T2, T3, T6**
R4. Facilitate the formation of community groups for pregnant women with assigned CHWs	**S1, S3, S4, O4, T1, T2, T6**
**2. Awareness Campaigns/Community Mobilization Sessions**	
R1. Conduct mobilization sessions and awareness campaigns at places of work (cross-border market, in the field, etc.) or community spaces (e.g. churches)	**S3, O1, W1, T1**
R2. Use radio programs and mobilization sessions to encourage men to participate in nutrition and health interventions	**W2, O4, T2**
R3. Design awareness campaigns that are recurring and recognizable (e.g. include logos, repeated taglines, images, posters)	**S1, S2, O2**
R4. Collaborate with local leaders (e.g. church leaders, community leaders) to promote messages of campaign in their spaces	**S3, W1, O1, T1, T2**
R5. Provide uniforms with recognizable logo to community facilitators or CHWs leading education sessions	**S2, O3, T2**
R6. Incorporate physical demonstrations into mobilization campaigns whenever possible	**S2, O5, T3**
**3. Early Childhood Development Centres**	
R1. Increase basic materials and improve management of home-based ECDs	**W2, O1, T3**
R2. Consult with caregivers on which skills or topics they want training on and provide them with incentives to attend trainings. Possible incentives could include transport allowances or free manuals and educational materials to take home.	**S3, W2, O2, O4, O6, O7, T4**
R3. Assign roles and responsibilities to parents, especially fathers, to increase involvement and contribution. Help parents set up Saving Lending Committees (SLCs) to pool money for ECD resources and porridge or milk programs. Create a transparency program in SLCs to ensure parents see how budget is managed and complement these groups with SBC campaign to explain importance of parent contribution to ECDs.	**S1, S2, S4, W1, W2, W3, W4, O1, O3, T1, T2, T3, T5, T6, T7, T8**

R4. Establish links between ECDs and Farmer Field Schools (FFS) for local procurement and agricultural education sessions for caregivers. This can be done through food system platform and farmer hubs.	**S1, S2, S3, W4, O2, T6**
R5. Ensure all centers engage in at-home follow-up with households to ensure that parents are adopting ECD lessons on healthy diets and lifestyle practices at home	**S2, O5, T8, T9**
**4. Exclusive Breastfeeding**	
R1. Encourage women to leave breastmilk at home when they go to work and educate other household members on proper feeding practices	**W2, O2, T3**
R2. Increase maternity leave (currently at 12 weeks)	**W2, T2, T3**
R3. Provide designated spaces for breastfeeding in workplaces and in community (e.g. at marketplaces)	**O3, T2, T3**
R4. Increase CHW and health centre supply of books and educational materials about breastfeeding and 1000 Days	**S1, O4, T1**
R5. Incorporate education about exclusive breastfeeding and proper maternal nutrition at community gatherings combined with growth monitoring (not just for women at ANC visits)	**S2, O1, O4, T1, T3**
**5. Farmer Field Schools**	
R1. Scale up SMS reminders for farmers to ensure guidance is followed after trainings and to encourage them not to sell their production	**S1, W1, W3, O2, T2, T5**
R2. Help in recruiting more FFS facilitators	**W3, O2, T2**
R3. Help in recruiting and training "model" or "example" farmers to pass on skills and educate community about nutrition (e.g. how to diversity meals, raise livestock, reserve animal products for home consumption)	**S2, S3, W1, W3, O2, T1, T2, T4, T6**
R4. Increase the number and frequency of trainings and provide regular refresher sessions for FFS facilitators to sustain their motivation and encourage continuous learning.	**S2, S3, W3, O3**
R5. Invest in establishing local feed industry and in research into new feed alternatives (e.g. rearing Black Soldier Flies as low-input low-cost option)	**W2, O1, T1, T3, T4, T6**
**6. Fruit Trees**	
R1. Work jointly with local government to invest in improved, modified seeds (require less inputs and land)	**W1, O2, O5, T1, T4**

R2. Phase out distributing seedlings to vulnerable families and replace with providing seedlings at a discounted price. Possibly include condition for future "payback" with fruit after a certain number of years	**O1, O6**
R3. Complement seedling distribution or discounted sales with a local mobilization campaign about importance of including fruits in the diet and incentivize keeping them for home consumption	**O3, O4, O5, T2**
R4. Help schools and ECDs find land in the community to plant fruit trees to supplement school feeding	**O3, T1**
**7. Kitchen Gardens**	
R1. Increase education sessions about best practices to grow vegetables on very small plots of land	**W1, O1, T1, T3**
R2. Provide seeds, bags, or soil needed to initiate kitchen gardens as incentives	**W1, O5, T1, T4**
R3. Scale up model plots and gardens in community (e.g. at ECDs) used for educational and training purposes. Complement this with awareness campaigns and mobilization about benefits of vegetables in the diet and reserving production for at-home consumption	**S1, S2, O1, O2, O3, O6, T1, T5**
R4. Assist families in accessing free or subsidized organic manure	**W1, O7, T1, T4**
R5. Encourage male participation in adopting and maintaining kitchen gardens and attendance at nutrition education sessions (e.g. men-targeted awareness campaigns at homes or places of work)	**S2, O6, T2, T6**
**8. NCD Prevention and Physical Activity**	
R1. Organize separate mass sport events or physical competitions for youth and adults, always complemented by education session	**S1, S2, S3, W1, O4, T1**
R2. Target women specifically to exercise through behavior change communication strategy which emphasizes the link to positive impact on children	**S1, S2, W1, W2, W3, O1, T1, T3, T4, T5, T6**
R3. Focus on setting women as examples in sports	**W2, W3, O1, O2, T3, T4, T5, T6**
R4. Incorporate physical activity interventions run by female teachers and education about healthy diet for NCD prevention into school feeding program	**S2, S3, W1, O2, O3, O4, T2**
R5. Incorporate physical activity and sports events targeting women into programs and activities that women already attend (e.g. health centre gatherings)	**S1, S2, W2, W3, O1, T2, T3, T4, T5, T6**
**9. Nutrition-Sensitive Direct Support (NSDS) and Shisha Kibondo**	

R1. Complement any monetary support with targeted education about family planning and/or facilitated access to contraceptives. Extend support until the child has reached 3 years of age instead of 2.	**S1, W1, O2, T1, T4**
R2. Base quantity of Shisha Kibondo provided on household size of beneficiary rather than on quantity needed for one child only	**W1, O2, T3**
R3. Initiate Shisha Kibondo at ECDs as an opportunity to teach parents how to administer it properly at home. Eventually transition to distribution at homes with close follow-up	**S1, W1, O1, O2, O3, T2, T3**
**10. School feeding program**	
R1. Encourage monetary and non-monetary parent contributions through mobilization and at-home visits	**S1, S2, W1, O1, O3, T1, T2, T4**
R2. Increase collaboration between parents, local leaders, and school teachers to help cultivate trust and support them in stablishing SLCs to pool money for resources to improve school feeding. Create a transparency program in SLCs to ensure parents see how budget is managed and complement these groups with SBC campaign to explain importance of parent contribution to school feeding	**S1, S2, W1, W2, O1, O2, T1, T2, T4, T5**
R3. Invest in building or finding designated eating spaces at schools (refectories) and in acquiring sufficient kitchen and cooking materials to meet demand	**O4, T3, T7**
R4. Establish links with Farmer Field Schools (FFS) for local procurement and agricultural education sessions (e.g. kitchen garden trainings)	**W1, W2, O2, O3, T4**
**11. Small Stock Distribution Program**	
R1. Replace or complement Girinka with small stock (chicken, rabbits, goats) using a sustainable business approach instead of distribution for free. Complement program with demand generation strategy to create consumer awareness about importance of animal products in the diet	**S2, O1, O4, T2**
R2. Combine livestock program with agroforestry initiatives (fruit seedlings at a discounted price)	**S2, O2, W2, T5**
R3. Involve entire family (youth especially) in trainings on livestock care	**W1, W2, T1**
R4. Invest in establishing local feed industry or in research into and adoption of new feed alternatives (e.g. rearing Black Soldier Flies as low-input low-cost option)	**W2, T5**
R5. Engage in close follow-up after distribution to ensure farmers are taking proper care of their livestock and to discourage them from selling	**W1, O3, T4**
R6. Help strengthen the veterinary system at the community level	**W1, W2, T6**
R7. Implement small stock distribution within structure of farmers hubs established by NICE project	**S3, O4, T2**

12. Trainings (CHWs and caregivers)	
R1. Consult with CHWs and caregivers on which trainings they want more of	**S2, S3, O1, O3, T1**
R2. Provide more refresher classes and consistent repetitions of trainings	**S3, W1, O2, O5, T2**
R3. Implement a form of exam or evaluation for CHWs to test their knowledge following trainings and/or provide a certification at the end of the training	**S1, S2, O3, O5, T1, T2**
R4. Increase materials used at trainings to promote long-term learning and review (e.g. books, brochures, take-home materials)	**S2, S3, W1, O3, O4, T1**
R5. Provide incentives for CHWs or caregivers to attend trainings given that they are not compensated (e.g. bus tickets for transportation, free manual or educative materials to take home)	**S3, W1, O3, O4, T1**
